# Morphological and Epidemiological Analysis of the Sphenoid Sinus Based on Computed Tomography and Magnetic Resonance Imaging: A Narrative Review

**DOI:** 10.3390/life16071105

**Published:** 2026-07-02

**Authors:** Kristian Bechev, Daniel Markov, Fares Ezeldin, Marin Kanarev, Aneliya Petrova, Elizabet Dzhambazova, Vladimir Aleksiev, Galabin Markov

**Affiliations:** 1 Department of Anatomy, Histology and Cytology, Faculty of Medicine, Medical University of Plovdiv, 4002 Plovdiv, Bulgaria; marin.kanarev@mu-plovdiv.bg (M.K.);; 2 Neurological Surgery, Pulmed University Hospital, 4000 Plovdiv, Bulgaria; 3 Department of General and Clinical Pathology, Medical University of Plovdiv, 4002 Plovdiv, Bulgaria; daniel.markov@mu-plovdiv.bg; 4 Department of Clinical Pathology, UMHAT “Pulmed”, 4002 Plovdiv, Bulgaria; 5 Department of Forensic Medicine and Deontology, Medical University of Plovdiv, 4002 Plovdiv, Bulgaria; fares.z.ezeldin@gmail.com; 6 Medical Oncology Ward, MHAT “Park Hospital”, 4002 Plovdiv, Bulgaria; 7 Department of Social Medicine and Public Health, Faculty of Public Healty, Medical University of Plovdiv, 4002 Plovdiv, Bulgaria; betidzhambazova@gmail.com; 8 Department of Thoracic Surgery, UMHAT “Kaspela”, 4002 Plovdiv, Bulgaria; vl_alex@abv.bg; 9 Department of Cardiovascular Surgery, Medical University of Plovdiv, 4002 Plovdiv, Bulgaria; 10 Faculty of Medicine, Medical University of Plovdiv, 4002 Plovdiv, Bulgaria; gabi_markov@abv.bg

**Keywords:** sphenoid sinus, pneumatization, anatomical variations, transsphenoidal surgery, skull base, computed tomography, magnetic resonance imaging, preoperative planning

## Abstract

Background: The sphenoid sinus occupies a strategically central position at the base of the skull, in close proximity to critical neurovascular structures including the pituitary gland, optic nerves, internal carotid arteries, and the cavernous sinus. Its complex anatomical configuration and pronounced individual variability render it a structure of paramount clinical importance in transsphenoidal approaches to the sellar and parasellar regions. Methods: A narrative review of the literature was conducted in accordance with the SANRA (Scale for the Assessment of Narrative Review Articles) guidelines for narrative reviews. A comprehensive search was performed across four electronic databases—PubMed/MEDLINE, Scopus, Web of Science, and Google Scholar—covering publications from January 2000 through March 2026, with the final search update completed in March 2026. An initial pool of 826 articles was identified. Following title and abstract screening, 421 articles were retained for full-text assessment; of these, 307 contributed to the conceptual synthesis, and 100 are directly cited in the manuscript. The literature selection process followed a structured flow diagram consistent with PRISMA reporting principles for identification and screening stages. Results: We synthesise current evidence on the embryological development, macroscopic and microscopic anatomy, morphological classifications, and epidemiological distribution of anatomical variations in the sphenoid sinus, with emphasis on radiological assessment and surgical relevance. The spectrum of pneumatization patterns—from the conchal type through presellar and sellar variants to the postsellar, clival, and lateral types—is reviewed alongside population-level prevalence data. Common and rare variations are examined, including intrasinus septation, Onodi cells, and bony dehiscences overlying the internal carotid artery and optic nerve. The comparative diagnostic capabilities of computed tomography and magnetic resonance imaging are evaluated, alongside emerging modalities such as three-dimensional reconstruction, virtual reality, and augmented reality. Conclusions: Detailed morphological knowledge of the sphenoid sinus plays an important role in surgical strategy, instrument selection, and risk mitigation in skull base surgery. Individualised preoperative imaging—integrating CT, MRI, and, where available, immersive digital technologies—is essential for safe transsphenoidal planning and cannot be replaced by standard anatomical templates.

## 1. Introduction

The sphenoid sinus (sinus sphenoidalis, SS) is an irregularly pneumatised cavity situated within the body of the sphenoid bone, occupying a central position at the base of the skull. As part of the paranasal sinus system, it holds particular clinical relevance in transsphenoidal surgical planning of the sellar and parasellar regions, owing to its close anatomical relationships with multiple vascular and neural structures, including the pituitary gland, the optic nerves, and the internal carotid arteries (ICA). On the basis of its anatomical location and topographical relationships with surrounding structures, the sphenoid sinus serves as a key anatomical landmark and surgical corridor in endoscopic and microscopic transsphenoidal approaches [1,2,3,4].

The development of the sphenoid sinus begins in early postnatal life and continues into adolescence. Its pneumatization follows a complex process, originating with the formation of ethmoid cells that gradually expand into the body of the sphenoid bone. Although pneumatization progresses over the years, it exhibits considerable individual variability [5,6,7,8]. Age-related changes, including incremental increases in sinus volume, may further influence both preoperative planning and intraoperative risk [9,10].

The walls of the sinus are in close anatomical contact with a number of critical neurovascular structures. Subtle morphological variations—including the degree of pneumatization, the number and position of intrasinus septa, the presence of Onodi cells, and bony dehiscences overlying adjacent vessels and nerves—may carry significant clinical and surgical implications [9,11,12,13]. These variations are not anecdotal; their distribution differs between populations and individuals, and their preoperative recognition is essential for risk stratification.

The clinical consequences of unrecognised anatomical variations at this level can be severe. Dehiscence or marked thinning of the bony wall overlying the cavernous segment of the internal carotid artery predisposes to catastrophic intraoperative haemorrhage, which remains one of the most feared complications of transsphenoidal surgery and carries significant morbidity and mortality even in experienced hands [9,11,14,15]. Proximity or direct contact between the optic nerve and the sinus mucosa—encountered in a clinically meaningful proportion of patients—increases the risk of iatrogenic visual loss, ranging from transient field defects to permanent blindness [14,15,16]. Pathological or surgical involvement of the pituitary gland may result in postoperative endocrine dysfunction, including panhypopituitarism or diabetes insipidus, while inadvertent entry into the cavernous sinus carries the additional risk of injury to cranial nerves III, IV, V_1_, V_2_, and VI, potentially producing diplopia, facial hypoaesthesia, or ophthalmoplegia [17,18]. The spread of inflammatory or infectious processes from the sphenoid sinus to adjacent intracranial compartments—including cavernous sinus thrombosis, meningitis, or epidural abscess—further underscores the clinical significance of detailed anatomical knowledge of this region [14,15].

The complex anatomy of the sphenoid sinus necessitates precise radiological assessment using computed tomography (CT) and magnetic resonance imaging (MRI), often combined with segmentation algorithms and three-dimensional reconstructions, in order to visualise spatial relationships and inform the selection of the optimal surgical corridor [1,2,19,20]. Three-dimensional CT reconstructions, in particular, enable clear differentiation of bony architecture from adjacent soft tissues [21,22,23]. Beyond standard preoperative imaging, virtual reality (VR) and augmented reality (AR) are increasingly being explored as adjuncts to surgical planning, offering immersive visualisation of patient-specific anatomy.

Precise morphological knowledge therefore plays an important role in surgical planning and risk reduction. The distance between the cavernous segments of the internal carotid arteries varies considerably across individuals and pathological conditions, requiring individualised assessment prior to any intervention; this has been demonstrated, among others, by Bechev et al. (2024) in a Bulgarian population based on a series of 112 MRI scans [24].

Despite an expanding body of literature, the available evidence on the sphenoid sinus remains fragmented across embryological, anatomical, radiological, and surgical perspectives, with substantial inter-population variability that complicates the formulation of universal morphological classifications. The present narrative review aims to synthesise current evidence on the morphological and epidemiological characteristics of the sphenoid sinus, with particular emphasis on its developmental anatomy, classification of pneumatization patterns, common and rare anatomical variations, comparative radiological assessment by CT and MRI, and emerging technologies for preoperative planning. By integrating these dimensions, the review seeks to provide a structured framework that supports surgical decision-making and risk mitigation in transsphenoidal approaches to the skull base.

## 2. Methods

This narrative review aimed to synthesise current evidence on the morphological and epidemiological characteristics of the sphenoid sinus, with emphasis on its developmental anatomy, pneumatization patterns, anatomical variations, radiological assessment, and surgical relevance.

### 2.1. Literature Search Strategy

A comprehensive literature search was conducted across four electronic databases: PubMed/MEDLINE, Scopus, Web of Science, and Google Scholar. The search was performed between February 2024 and March 2026, with the final search update completed in March 2026. Publications dated from January 2000 through March 2026 were considered for inclusion. Earlier seminal works of historical, embryological, or conceptual relevance (Stanek, 1977; Gould and Lewontin, 1979; Moss, 1997; Sirikci et al., 2000 [11,25,26,27]) were retained where they provided foundational context for the discussion.

The following keywords and Medical Subject Headings (MeSH) terms were used, individually or in combination with Boolean operators (AND, OR): “sphenoid sinus”, “pneumatization” OR “pneumatization”, “sphenoid sinus anatomy”, “sphenoid sinus variations”, “intrasinus septa”, “Onodi cell”, “transsphenoidal surgery”, “endoscopic endonasal approach”, “skull base anatomy”, “computed tomography” AND “sphenoid sinus”, “magnetic resonance imaging” AND “sphenoid sinus”, “preoperative planning”, “virtual reality” AND “endonasal surgery”, “augmented reality” AND “skull base”.

The reference lists of included articles were additionally screened by hand to identify relevant publications not retrieved through the initial database search (citation tracking).

### 2.2. Inclusion and Exclusion Criteria

Articles were considered eligible for inclusion if they:

➢ addressed the embryology, anatomy, morphometry, or epidemiology of the sphenoid sinus;
➢ reported CT- or MRI-based assessment of sphenoid sinus pneumatization, septation, or surrounding neurovascular relationships;
➢ discussed surgical implications of sphenoid sinus anatomy for transsphenoidal or extended endoscopic endonasal approaches;
➢ were peer-reviewed original research articles, anatomical or radiological studies, case series, or narrative and systematic reviews;
➢ were published in English.

Articles were excluded if they:

➢ focused exclusively on pathological lesions (neoplastic, inflammatory, or infectious) without addressing anatomical variation;
➢ were available only as conference abstracts, editorials, opinion pieces, or non-peer-reviewed sources;
➢ were duplicates, or did not provide sufficient anatomical or methodological detail.

### 2.3. Selection Process and Data Extraction

**Figure 1:**
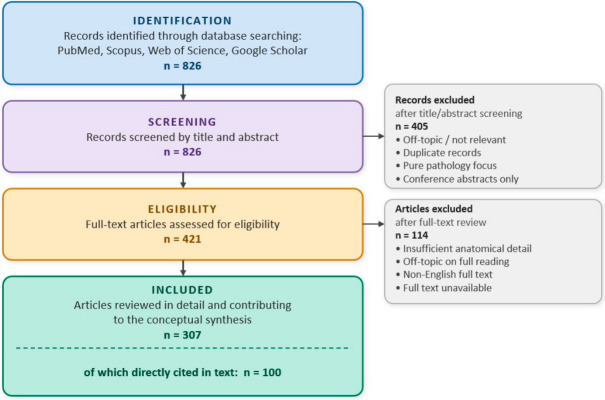
Flow diagram of the literature search and selection process, illustrating the four stages (Identification, Screening, Eligibility, Inclusion) and the reasons for exclusion at each step. Schematic flow diagram—not to scale; intended for methodological transparency.

An initial pool of 826 articles was identified through the database search. Following removal of duplicates and screening of titles and abstracts against the eligibility criteria, 421 articles were retained for full-text evaluation. Of these, 307 articles were assessed in detail and contributed to the conceptual synthesis of the present review; of these, 100 are directly cited in the manuscript text (Figure 1).

The literature search and selection process was conducted by the primary author (K.B.), with independent verification of eligibility applied to a representative sample of articles by a second author (D.M.). Disagreements regarding inclusion were resolved through discussion and consensus between the two reviewers. No formal inter-rater reliability coefficient was calculated, in accordance with standard practice for narrative reviews.

The 211 articles that contributed to the conceptual synthesis but are not directly cited in the manuscript text served to inform the broader interpretive framework of the review—including the contextualisation of prevalence data, the identification of methodological trends across the literature, and the formulation of clinical implications—without providing specific data points or statements that required direct attribution. This approach is consistent with the narrative review methodology, in which the synthesis of a large body of literature necessarily informs the intellectual framework of the manuscript beyond what can be individually cited within the constraints of the text.

Information extracted from the selected articles included: embryological and developmental data; morphometric measurements (linear dimensions, intersinus distances, wall thickness, intercarotid distance); classification systems of pneumatization (Hamberger, Güldner, Wang, Vaezi, Bilgir); prevalence of anatomical variants across different populations; imaging characteristics on CT and MRI; and surgical considerations, including the application of three-dimensional reconstruction, virtual reality, and augmented reality.

The included publications comprised 51 original research articles (including anatomical, radiological, and morphometric studies), 27 systematic reviews or meta-analyses (including four systematic reviews on the application of virtual and augmented reality in transsphenoidal surgery 97–100), 14 narrative reviews, and 8 book chapters or reference anatomical works, with the remaining sources representing case series, technical reports, and methodological papers. Findings were synthesised thematically rather than statistically, in accordance with the narrative review methodology. As the present review did not involve human subjects or the generation of original empirical data, no ethical approval was required, and no formal quantitative pooling or risk-of-bias assessment was undertaken.

## 3. Results

### 3.1. Evolution and Development of the Sphenoid Sinus

#### 3.1.1. Evolution and Functional Hypotheses of the Paranasal Sinuses

The evolution of the paranasal sinuses remains one of the most debated questions in comparative cranial anatomy. Analogous pneumatic structures occur across many animal species, but their expression varies considerably within and between taxa. The sphenoid sinus is present in all apes, whereas the ethmoid and frontal sinuses occur only in African apes and humans. The mechanisms driving these patterns remain incompletely understood, and the unpredictable distribution of sinuses across extant species limits their utility for reconstructing past phylogenetic patterns [13,25,26,27,28,29,30]. Several functional hypotheses have been proposed to explain the existence of the paranasal sinuses, including reduction in skull weight, nitric oxide (NO) production, and equalisation of intracranial and external pressures. Among these, the role of NO as a biologically active mediator produced within the paranasal sinus mucosa has attracted considerable interest. NO is synthesised from L-arginine by a family of nitric oxide synthase (NOS) enzymes, of which three isoforms have been identified in the respiratory mucosa: neuronal NOS (nNOS, type I), inducible NOS (iNOS, type II), and endothelial NOS (eNOS, type III). In the sinonasal epithelium, constitutively expressed eNOS and nNOS are responsible for baseline NO production, while iNOS—which is upregulated in response to inflammatory stimuli, bacterial products, and cytokines—mediates substantially higher NO output during infectious or inflammatory states [31,32,33].

The physiological significance of NO produced within the paranasal sinuses is multifaceted. NO enhances mucociliary clearance by stimulating ciliary beat frequency in the pseudostratified respiratory epithelium, thereby supporting the active transport of mucus and particulate matter towards the ostium [31,34,35]. NO exerts direct vasodilatory effects on the submucosal microvasculature through activation of soluble guanylate cyclase and elevation of cyclic GMP, contributing to the regulation of local blood flow and mucosal perfusion [32,33]. NO possesses potent antimicrobial properties: at the concentrations generated within sinus cavities, it inhibits bacterial replication, disrupts biofilm formation, and impairs viral replication, thereby contributing to innate mucosal defence [31,32,33]. The sphenoid sinus, owing to its enclosed anatomical position and relatively small ostium, may generate particularly high intraluminal NO concentrations, which are then delivered to the lower respiratory tract during nasal breathing—a mechanism proposed to contribute to pulmonary vasodilation and ventilation-perfusion matching [32,36,37].

Beyond NO, there is emerging evidence that the sinonasal mucosa may produce other gaseous signalling molecules—gasotransmitters—including hydrogen sulphide (H_2_S) and carbon monoxide (CO). H_2_S, synthesised primarily by cystathionine-β-synthase (CBS) and cystathionine-γ-lyase (CSE) in airway epithelial and smooth muscle cells, has been shown to exert cytoprotective, anti-inflammatory, and vasodilatory effects in the respiratory mucosa, and may modulate mucociliary function. CO, produced endogenously by haem oxygenase (HO-1 and HO-2), similarly possesses anti-inflammatory and vasodilatory properties and has been detected in exhaled nasal air. Although the specific contribution of these gasotransmitters to the physiology of the sphenoid sinus remains incompletely characterised, their recognised roles in the broader sinonasal system suggest that the functional biology of the paranasal sinuses extends well beyond the better-studied NO pathway [31,32,33].

More rigorous analyses incorporating soft-tissue mass, however, have shown that the weight-reduction effect is negligible, while other proposed functions remain empirically contested [31,32,33]. Since the seminal critique of Gould and Lewontin (1979) [26], evolutionary biology has emphasised that not all morphological traits represent direct adaptations; some emerge as developmental or geometric consequences of selection acting on adjacent structures [38,39]. This consideration is particularly relevant to the sphenoid sinus, whose pneumatization may reflect not direct selective pressure but the developmental dynamics of the surrounding cranial base.

#### 3.1.2. Development of the Craniofacial Region

The morphology of the sphenoid sinus cannot be considered in isolation from the development of the cranial base, with which it shares both spatial and developmental origins. The developmental trajectory of the neurocranium—particularly the basis cranii — is largely a response to brain enlargement during ontogeny [40,41,42]. The cranial base contains major growth centres within its synchondroses, and its development simultaneously drives facial growth [13,38]. Because the sphenoid sinus is anatomically embedded within this developmental space, its size, orientation, and pattern of pneumatization are influenced by the same forces that shape the surrounding skull base.

The ontogenetic study by Neubauer et al. (2010) [43] demonstrated that the shape of the human basis cranii interna is uniquely associated with perinatal head shape and contributes to many craniofacial features specific to humans. Differences in the internal cranial surface observed across primates emerge during early ontogenesis, when brain growth rates are highest and before the cranial bones have fully ossified, supporting the view that brain growth is the principal driver of neurocranial shape [29,38]. The underlying mechanisms involve expression of genes signalling dural growth: as the expanding brain presses against the inner cranial base, growth factors are stimulated in both the calvaria and the skull base [40,44].

Because the cranial base supports the brain and transmits cranial nerves, vessels, venous sinuses, the brainstem, and the pituitary gland, it is under stricter genetic control than other regions of the skull. This manifests as accelerated endochondral ossification, with the basis cranii reaching adult dimensions before the rest of the craniofacial skeleton [45,46]. Experimental mouse models indicate that approximately 87% of basal cranial angulation can be accounted for by brain size, neurocranial dimensions, and facial size [6,45]. The midline and lateral compartments of the cranial base behave semi-independently—the midline contributing to angulation of the basis cranii in response to encephalisation, the lateral influencing facial shape and orientation [47,48]. The sphenoid bone sits at the junction of these compartments, placing the sphenoid sinus at a developmentally pivotal location with direct implications for transsphenoidal surgical planning.

#### 3.1.3. Embryonic Development of the Sphenoid Bone

The sphenoid bone develops from seven paired ossification centres, predominantly through endochondral bone growth, with only minimal foci of membranous ossification [25,49,50]. The ossification of the greater wing and the lateral pterygoid plate begins between the foramen ovale and the foramen rotundum during the 8th week of gestation (alisphenoidale). One week later, during the 9th week, a separate ossification centre appears on the lateral aspect of the optic canal, corresponding to the lesser wing (orbitosphenoidale) [25,50,51,52].

The body of the sphenoid bone ossifies from two pairs of centres arranged in anteroposterior sequence. The posterior pair (basisphenoidale) develops during the 3rd month of gestation in the floor of the sella turcica, while the anterior pair, appearing later, forms the preclinoid region. A small punctiform ossification develops lateral to the basisphenoidale for the lingula of the sphenoid bone and the adjacent portion of the carotid sulcus, which subsequently fuses with the basisphenoidale. The ossification centres of the basisphenoidale fuse with each other during the 4th month of fetal development, and the ossification centres of the lesser wing fuse with those of the sphenoid body bilaterally at the same time. The two halves of the sphenoid body, together with the basisphenoidale, fuse only by the 8th month, while the cartilage between the anterior and posterior pairs regresses before or shortly after birth [9,25,50,51].

An additional ossification centre on the medial portion of the pterygoid process appears during the 2nd month of embryonic development via membranous ossification, as does a smaller centre at the tip of the greater wing. All other centres develop endochondrally. At birth, the sphenoid bone consists of three parts: a central component (formed from already-fused ossification centres) and two lateral components, each containing the greater wing and the pterygoid process. These three parts, initially connected by a thin layer of cartilage, fuse during the first postnatal year [25,38,51]. The cavities of the sphenoid bone begin to develop from the primary skull through several ossification centres starting in the 5th month of gestation, setting the stage for subsequent pneumatization of the sinus [13,30,40].

#### 3.1.4. Embryonic Development of the Sphenoid Sinus

The sphenoid sinus (sinus sphenoidalis) begins to develop around the 3rd–4th month of gestation, through bilateral invagination of the nasal mucosa into the body of the sphenoid bone. These mucosal invaginations form depressions (sphenopalatine fossae) located adjacent to the future openings of the sphenoid sinus. At the time of birth, the sphenoid sinuses are present but not yet aerated, with a total volume not exceeding 2 mm^3^—comparable in size to a small pea—representing cavities continuous with the spheno-ethmoidal recesses [25,40,50,51,53].

Pneumatization of the sphenoid sinus is a complex, stepwise process spanning prenatal development through the end of puberty, best understood as a sequence of morphological phases. During intrauterine life, between the 3rd and 4th gestational months, mucosal invagination of the cartilaginous retrocapsular region of the future nasal cavity establishes a recess between the presphenoidal body and the middle nasal concha [13,54,55]. At this stage no air space exists; the recess is occupied by embryonic connective tissue surrounded by cartilage. At birth, a small recess is identifiable but lacks the volume necessary for pneumatization, and the prospective sinus space remains filled with bone marrow substrate or undifferentiated cartilaginous tissue.

During the first months of postnatal life, the prospective sinus remains filled with red bone marrow, which gradually undergoes conversion to yellow (adipose) bone marrow [56,57,58]. This conversion proceeds in an anteroposterior direction, beginning in the anterior part of the sphenoid body [59,60]. The transition from red to yellow marrow is a necessary precursor to true pneumatization and must occur before active cavitation can begin.

The period between the 7th postnatal month and approximately the 2nd year of life marks the onset of active cavitation: the fatty bone marrow in the anterior region is resorbed, freeing space for air cells to enter through continued mucosal invagination from the nasal cavity [38,61,62,63]. The initial trajectory of air-cell expansion is anterocaudal, towards the basisphenoid plate. During childhood, pneumatization progresses slowly along well-documented directions: the air cells originate at the ostium and expand posteriorly, inferiorly, and laterally. This pattern is critical for predicting the final morphological configuration—caudal expansion tends to produce the clival type, while lateral expansion may thin the bony layer overlying the internal carotid artery or the optic canal.

By early adolescence (12–14 years), the sphenoid sinus reaches close to its final volume of approximately 6 cm^3^ combined for both halves, although subtle micromorphological changes may continue beyond puberty [13,61]. The term “mature” pneumatization refers to a stable internal volume with fixed relationships between intra-sinus features—carotid and optic canals, septa, and recesses. The overall process may extend caudally to the base of the occiput by the time of ossification of the spheno-occipital synchondrosis, around age 20.

The development of the sphenoid sinus can be conceptualised in two principal stages:

➢ Primary pneumatization—formation of a depression in the spheno-ethmoidal fossa, from the neonatal period to approximately 4 years of age.
➢ Secondary pneumatization—expansion of connective tissue into the skeletal framework of the viscerocranium, beginning around 4 years and completing between 12 and 16 years of age.

**Figure 2:**
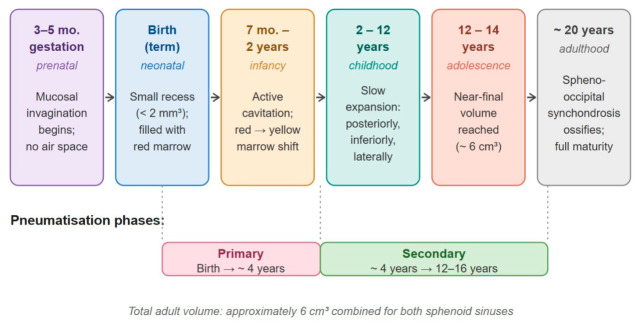
Timeline of embryological development of the sphenoid sinus, from initial mucosal invagination at 3–5 months of gestation to full maturation at approximately 20 years of age. The two principal pneumatization phases—primary (birth to ~4 years) and secondary (~4 to 12–16 years)—are indicated below the milestones. Schematic representation—not to scale; intended for didactic purposes and not reflecting precise chronological proportions in all individuals.

The principal milestones of this developmental sequence, together with the two pneumatization phases, are summarised in Figure 2.

Inter-individual variation in this process is substantial. In some individuals, pneumatization effectively ceases early, producing the conchal type with minimal air-space formation. In others, expansion is extensive, reaching the anterior clinoid process, the greater wings, and even the pterygoid process—corresponding to the sellar and postsellar types [49,64,65]. Such variations are clinically significant: in cases of extreme pneumatization, the bony walls may become thin or even absent over critical neurovascular structures, constituting a fundamental factor in surgical planning. The direction and degree of pneumatization may also be asymmetrical. The septum dividing the sinus rarely lies precisely on the midline; it often terminates on bony protrusions such as the carotid sulcus or the optic canal [66,67]. Consequently, development in each half may follow a different temporal pattern, with one side reaching maturity considerably earlier than the other, and in some individuals more than one intersinus septum may form before growth ceases.

An alternative developmental hypothesis was proposed by Jankowski et al. (2016) [36], who suggested that pneumatization of the sphenoid sinus is not only driven but actually initiated by bone metabolism and remodelling of the bone marrow, which constitutes the determining step for cavity formation. In this view, the sphenoid sinus is formed through primary bone cavitation, with nitric oxide produced within the cavity as a consequence of bone marrow involution and its subsequent evacuation through connecting openings to the nasopharyngeal complex—described as functional “chimneys” [36,37]. According to this hypothesis, the sphenoid sinus possesses a distinct initial developmental stage that begins and concludes considerably earlier than that of the other paranasal sinuses [36,56,58].

From a surgical standpoint, the phasic nature of pneumatization imposes strict considerations of patient age in transsphenoidal access planning. In young children, surgical approaches through an incompletely pneumatised sinus carry risks due to both the limited working space and the thick surrounding bone [14,15,68]. Adequate operative manoeuvrability becomes available only once mature volume is established during adolescence.

### 3.2. Types of Pneumatization

**Figure 3:**
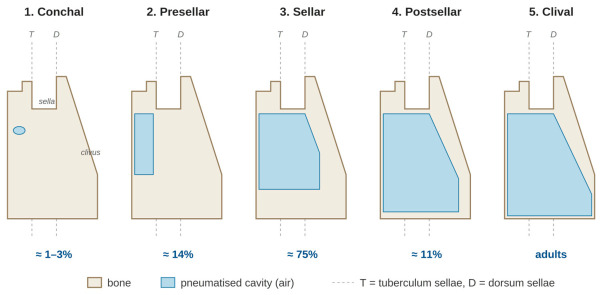
Schematic sagittal representation of the five principal types of sphenoid sinus pneumatization according to the Hamberger classification. The blue regions indicate the extent of pneumatization within the body of the sphenoid bone; reference lines T (tuberculum sellae) and D (dorsum sellae) define the boundaries used in the classification. Percentages indicate the approximate prevalence in the general population, as reported across multiple CT-based cohort studies [14,69,70,71].

Multiple morphological classifications are based on the position and depth of the pneumatised recess relative to the body of the sphenoid bone. Five principal types are recognised (Figure 3), with an additional agenetic variant proposed by some authors.

The most widely cited classification, originally described by Hamberger et al. (1961) [69] is based on the position and depth of the pneumatised recess relative to the body of the sphenoid bone. Five principal types are recognised, with an additional agenetic variant proposed by some authors (characterised by the complete absence of pneumatization):

➢ Conchal type—The body of the sphenoid bone is poorly developed and remains predominantly solid. Only a small invagination is detectable below the sella turcica, forming a flattened sphenoid recess in the spheno-ethmoidal fossa without contact with the body of the sphenoid bone. Reported in approximately 1–3% of cases [14,69,70];
➢ Presellar type—Pneumatization of the sphenoid body does not extend beyond the vertical line passing through the tuberculum sellae; below this level the bone remains fully compact. Reported in approximately 14% of cases [14,69,70];
➢ Incomplete sellar type—Pneumatization reaches the floor of the sella turcica but does not extend beyond the vertical line through the dorsum sellae [69,70,71];
➢ Sellar type—Pneumatization reaches the upper third of the clivus and may involve adjacent anatomical structures. This is the most common type, observed in approximately 75% of cases, and is the most clinically significant for transsphenoidal access. Some authors further divide it into ‘incomplete’ and “complete” subtypes [69,71,72].
➢ Postsellar type—Extensive pneumatization reaching the middle third of the clivus and the pterygoid process of the sphenoid bone, observed in approximately 11% of cases [15,69,71].

An alternative classification categorises pneumatization of the sphenoid bone along an anteroposterior axis:

➢ Anterior—absence of presellar or sellar pneumatization.
➢ Posterior—presence of sellar, postsellar, or clival pneumatization [16,71,72,73,74].

A further classification, is based on the degree of development of the pterygoid (PE), lateral (LE), and clival (CR) recesses. The clival recess serves as the principal parameter for the degree of pneumatization, while the lateral and pterygoid recesses are particularly informative when planning extended transsphenoidal approaches:

➢ Type 0—sellar pattern with absent lateral and pterygoid recesses; the sinus is not fully pneumatised and the bone remains relatively thick.
➢ Type 1—postsellar pattern with limited pneumatization; the lateral recesses extend below the level of the foramen rotundum, while the pterygoid recesses are absent.
➢ Type 2—full pterygoid pneumatization, in which the pterygoid recesses extend below the level of the vidian canal and the lateral recesses extend anterior to the lateral aspect of the foramen rotundum. The lateral recesses are well developed and extend posteriorly along the line connecting the V2 and V3 branches of the trigeminal nerve.
➢ Type 3—pterygoid pneumatization reaching its maximum extent, with the pterygoid recesses extending fully to the pterygoid wings and lateral pterygoid plates. The internal carotid arteries, as well as cranial nerves V and VI, may protrude into the sinus on either side [1,2,3,4,5,6,7,8,9,10,11,12,13,16,19,20,21,22,23,24,25,71,72,73,74].

The morphological type of pneumatization evolves with age. The conchal type predominates during the first 3 years of life, the presellar type between approximately 3 and 7 years, the sellar type between 8 and 12 years, and the postsellar type in late adolescence and adulthood. The clival type, representing the most extensive pneumatization pattern, is found exclusively in adults and is associated with maximal expansion throughout the body of the sphenoid bone and into the basilar part of the occipital bone [14,15,16,71].

Clinical significance of pneumatization types.

The morphological type of sphenoid sinus pneumatization is not merely a descriptive anatomical parameter; it carries direct and measurable consequences for surgical planning, intraoperative risk, and the selection of operative approach in transsphenoidal and extended endoscopic endonasal procedures [14,15,71].

The conchal and presellar types, characterised by limited or absent pneumatization of the sphenoid body, present the greatest technical challenge in transsphenoidal access. The absence of an adequately developed air space requires extensive drilling of the sphenoid body before the sellar floor can be reached, substantially increasing operative time, the risk of disorientation, and the likelihood of injury to adjacent neurovascular structures—particularly in the context of limited intraoperative landmarks [14,15]. Neuronavigation and intraoperative fluoroscopy are especially important adjuncts in these cases.

The sellar type—by far the most prevalent, encountered in approximately 75% of adults—provides the optimal conditions for standard transsphenoidal access. The well-pneumatised sinus offers clear anatomical landmarks, adequate working space, and a direct corridor to the sellar floor, which underlies the widespread adoption of the endoscopic endonasal transsphenoidal approach as the standard of care for pituitary pathology [14,69,71]. However, even within the sellar type, variability in the degree of lateral pneumatization and the configuration of intrasinus septa may influence the accessibility of parasellar structures and the risk of vascular injury.

The postsellar and clival types, representing the most extensive pneumatization patterns, are associated with maximal expansion of the sinus into the clivus and, in some cases, into the pterygoid processes and greater wings. While these types facilitate wide surgical exposure—particularly in extended approaches targeting the upper clivus, the petroclival region, or the cavernous sinus—they are paradoxically associated with an increased risk of neurovascular complications. Progressive pneumatization thins the bony coverage over the cavernous segment of the internal carotid artery and the optic nerve, increasing the frequency of dehiscences and the likelihood of inadvertent vascular or neural injury [14,15,16]. Additionally, in highly pneumatised sinuses the intersinus septum frequently deviates laterally and terminates on the carotid sulcus, creating a particular hazard during septal manipulation [11,14,75].

The lateral and pterygoid recesses, assessed by the Type 0–3 classification of recess development, are of particular relevance in extended endoscopic approaches to the cavernous sinus, the foramen rotundum, and the vidian canal. Well-developed lateral recesses (Types 2 and 3) provide direct surgical access to these structures but simultaneously place cranial nerves V_2_, V_3_, and VI, as well as the internal carotid artery, in immediate proximity to the operative field [1,15,71]. Preoperative CT assessment of recess morphology is therefore an indispensable component of surgical planning in extended skull base approaches [1,2,19,76].

### 3.3. Anatomy and Morphology of the Sphenoid Bone and Sphenoid Sinus

#### Macroanatomy of the Sphenoid Bone

**Figure 4:**
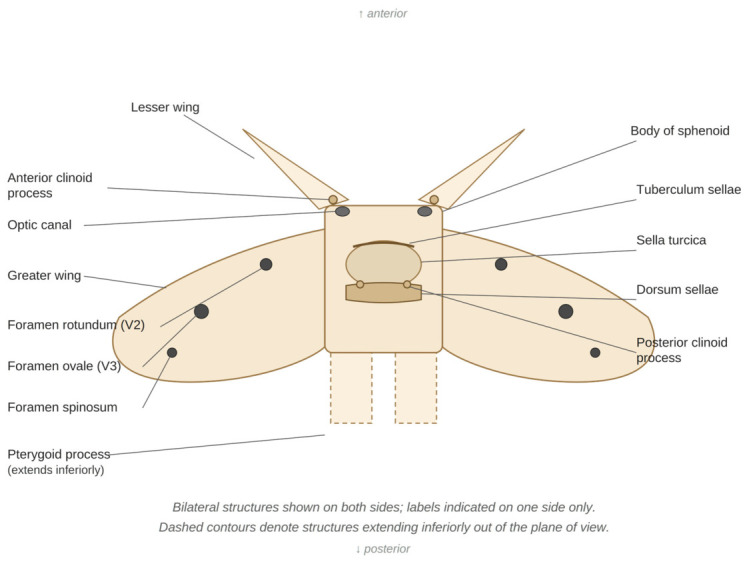
Schematic superior view of the sphenoid bone illustrating its principal anatomical components. The body of the sphenoid contains the sella turcica, bounded anteriorly by the tuberculum sellae and posteriorly by the dorsum sellae with its posterior clinoid processes. The lesser wings project anteriorly from the body and bear the anterior clinoid processes and the optic canals. The greater wings extend laterally and contain three principal foramina—*foramen rotundum* (V2), *foramen ovale* (V3), and *foramen spinosum*. The pterygoid processes are indicated by dashed outlines, as they extend inferiorly out of the plane of view. Bilateral structures are shown on both sides; labels are indicated on one side only. Schematic representation—not to scale; the figure is intended for didactic purposes and does not reflect precise anatomical proportions.

The sphenoid bone (*os sphenoidale*) is among the most morphologically complex bones of the human skull, occupying a strategically central position at the base of the cranium. It articulates with eight other bones and serves as the structural link between the neurocranium and the facial skeleton. Anatomically, it consists of a central body and several paired lateral processes—two greater wings, two lesser wings, and two pterygoid processes—that extend laterally and inferiorly [50,73,74]. A schematic representation of the principal components of the sphenoid bone is provided in Figure 4.

Body of the sphenoid bone. The body (*corpus ossis sphenoidalis*) is irregularly cuboidal in shape and houses the central pneumatised space—the sphenoid sinus. The superior surface bears the sella turcica, a saddle-shaped depression containing the hypophyseal fossa, which accommodates the pituitary gland. The anterior boundary of the sella turcica is marked by the tuberculum sellae, while its posterior boundary is formed by the dorsum sellae, which terminates in two projections—the posterior clinoid processes (*processus clinoidei posteriores*). The clivus (*clivus ossis sphenoidalis*) is the bony slope located behind the dorsum sellae and articulates posteriorly with the basilar part of the occipital bone via the spheno-occipital synchondrosis [2,24,73].

Greater wings. The greater wings (*alae majores*) form part of the floor of the middle cranial fossa and articulate with the temporal, parietal, frontal, and zygomatic bones. They are traversed by three principal foramina:

➢ the foramen rotundum, transmitting the maxillary nerve (V2);
➢ the foramen ovale, transmitting the mandibular nerve (V3);
➢ the foramen spinosum, transmitting the middle meningeal artery (*a. meningea media*).

The temporal surface contributes to the temporal fossa; the orbital surface forms part of the lateral wall of the orbit; and the maxillary surface participates in the formation of the pterygopalatine fossa [1,3,74].

Lesser wings. The lesser wings (*alae minores*) are smaller, triangular projections that form the posterior boundary of the anterior cranial fossa and the superior boundary of the superior orbital fissure. Each lesser wing contains the optic canal (*canalis opticus*), through which the optic nerve (*n. opticus*) and the ophthalmic artery (*a. ophthalmica*) pass. Posteriorly, each lesser wing terminates in the anterior clinoid process (*processus clinoideus anterior*), an important landmark in skull base neurosurgery [1,2,73].

Pterygoid processes. The pterygoid processes (*processus pterygoidei*) descend from the body of the sphenoid bone and each consist of two laminae—medial and lateral—separated by the pterygoid fossa. The medial lamina terminates in the pterygoid hamulus, around which passes the tendon of the *m. tensor veli palatini*. The pterygoid canal (*canalis pterygoideus*, also known as the vidian canal), transmitting the nerve of the pterygoid canal (*nervus canalis pterygoidei*, or vidian nerve), traverses the base of the pterygoid process and serves as a key anatomical landmark in extended endoscopic endonasal approaches [15,73,74].

### 3.4. Macroanatomy of the Sphenoid Sinus

The sphenoid sinus (*sinus sphenoidalis*) is a paired, pneumatised cavity located within the body of the sphenoid bone (*corpus ossis sphenoidalis*). As part of the paranasal sinus system, it occupies the most posterior position among the pneumatic spaces of the skull [3,17,77,78]. Its close anatomical relationship with vital intracranial structures—including the pituitary gland (*glandula pituitaria*), the optic nerves (*nervi optici*), the internal carotid arteries (*arteriae carotides internae*), and the cavernous sinuses (*sinus cavernosi*)—defines its central role in transsphenoidal surgical approaches [3,17,77,78].

Shape and septation. The sphenoid sinus is irregular in shape and exhibits considerable inter-individual variability. In the typical adult, the cavity is divided into asymmetrical right and left halves by an intersinus septum (*septum sinuum sphenoidalium*). This septum is frequently deviated from the midline and often terminates at the carotid sulcus (*sulcus caroticus*) or at the bony wall overlying the internal carotid canal (*canalis caroticus*), thereby increasing the risk of vascular injury during transsphenoidal manipulation [11,14,75,79].

Walls and ostium. The walls of the sphenoid sinus are composed of compact bone of variable thickness. The anterior wall faces the spheno-ethmoidal recess (*recessus sphenoethmoidalis*) and contains the sphenoid ostium (*apertura sinus sphenoidalis*)—an oval or rounded opening that establishes communication between the sinus and the nasal cavity [80,81].

Size and volume. The dimensions of the sphenoid sinus vary considerably between individuals, depending principally on the degree of pneumatization. The average adult volume ranges from approximately 6 to 9 cm^3^ combined for both halves [13,14,82], although in cases of extensive pneumatization the total volume may exceed 14 cm^3^ [15,16,83].

Topographic relations. The sphenoid sinus is anatomically related to multiple critical structures in all directions:

➢ Superior: the optic chiasm, the cavernous sinus, the pituitary gland, and the intracranial portion of the optic nerve;
➢ Inferior: the choanae and the nasopharynx;
➢ Lateral: the cavernous sinus, the internal carotid artery, the optic nerve, and the maxillary branch of the trigeminal nerve (V2);
➢ Medial: the contralateral half of the sinus, separated by the intersinus septum;
➢ Posterior: the clivus and the brainstem;
➢ Anterior: the ethmoidal air cells and the superior part of the nasal septum.

The sphenoid sinus communicates with the nasal cavity through the sphenoid ostium, which opens into the spheno-ethmoidal recess [78,80,81].

### 3.5. Microanatomy

The mucosal lining of the sphenoid sinus is morphologically similar to that of the rest of the upper respiratory tract. It consists of a pseudostratified ciliated columnar epithelium overlying a basement membrane, supported by a layer of loose connective tissue (lamina propria) that contains numerous seromucous glands [7,56,80,84]. The ciliary apparatus mediates mucociliary clearance—the active transport of mucus, cellular debris, and pathogens towards the sphenoid ostium and, from there, into the nasal cavity [34,35,85]. The regulation of mucociliary clearance in the sphenoid sinus is further modulated by nitric oxide (NO), produced locally within the sinonasal mucosa via constitutive and inducible NOS isoforms, as discussed in Section 3.1.1.

The mucosa of the sphenoid sinus is thinner than that of the other paranasal sinuses, a feature that has been proposed to be associated with a lower incidence of inflammatory diseases, though direct comparative evidence for this association remains limited [86,87,88]. At the same time, this reduced thickness renders the lining more susceptible to mechanical injury, including during surgical instrumentation [57,81,89].

Vascularisation. The sphenoid sinus receives its arterial supply principally from branches of the maxillary artery (*a. maxillaris*) and the ophthalmic artery (*a. ophthalmica*). Venous drainage occurs predominantly via the pterygoid venous plexus (*plexus venosus pterygoideus*) [35,73,74].

### 3.6. Molecular and Cellular Biology of the Sphenoid Sinus Mucosa

The functional biology of the sphenoid sinus extends well beyond its macroscopic and microscopic anatomy. At the cellular and molecular level, the sinonasal mucosa is a dynamic interface between the external environment and the intracranial compartment, mediating innate immune defence, mucosal homeostasis, and local neuroendocrine signalling through a complex network of interacting cell types and molecular pathways [34,35,85].

#### 3.6.1. Epithelial Cell Biology and Mucociliary Function

The pseudostratified ciliated columnar epithelium lining the sphenoid sinus comprises several functionally distinct cell populations: ciliated columnar cells, goblet cells, basal cells, and brush cells. Ciliated cells—the predominant cell type—bear approximately 200–300 motile cilia per cell, each driven by dynein motor proteins in a coordinated metachronal wave pattern that propels the overlying mucus layer towards the sphenoid ostium at a rate of approximately 5–8 mm per minute under physiological conditions [34,35]. Goblet cells contribute to the two-layered mucus system: a low-viscosity periciliary layer (sol phase) immediately surrounding the cilia, and a more viscous gel layer (gel phase) that traps particulate matter and microorganisms. The composition and rheological properties of this mucus bilayer are regulated by the balance of secretion from goblet cells and seromucous glands, and by transepithelial ion transport—principally through the cystic fibrosis transmembrane conductance regulator (CFTR) and epithelial sodium channels (ENaC), which together govern the hydration state of the periciliary layer [34,35,85].

Basal cells serve as the principal stem cell population of the respiratory epithelium, giving rise to both ciliated and secretory cell lineages during normal turnover and following epithelial injury. Their activation is regulated by Notch signalling pathways and by local cytokine gradients, particularly those involving transforming growth factor-β (TGF-β) and epidermal growth factor (EGF) [35,85]. Disruption of basal cell function—as may occur in chronic inflammatory states—results in impaired epithelial regeneration, squamous metaplasia, and progressive loss of mucociliary competence.

#### 3.6.2. Innate Immune Mechanisms

The sinonasal epithelium constitutes the first line of innate immune defence against inhaled pathogens, allergens, and environmental irritants. Pattern recognition receptors—including Toll-like receptors (TLRs), NOD-like receptors (NLRs), and RIG-I-like receptors—are expressed on the apical surface and within the cytoplasm of epithelial cells, enabling rapid detection of pathogen-associated molecular patterns (PAMPs) and damage-associated molecular patterns (DAMPs) [35,85]. Activation of these receptors initiates a cascade of downstream signalling events, principally through the NF-κB and MAPK pathways, resulting in the upregulation of pro-inflammatory cytokines—including interleukin-1β (IL-1β), interleukin-6 (IL-6), interleukin-8 (IL-8/CXCL8), and tumour necrosis factor-α (TNF-α)—and the recruitment of innate immune effector cells, including neutrophils, macrophages, and natural killer cells [35,85,86,87].

In addition to cytokine-mediated signalling, the sinonasal epithelium produces a range of antimicrobial peptides—including defensins, lactoferrin, lysozyme, and secretory immunoglobulin A (sIgA)—that constitute a non-specific chemical barrier against microbial colonisation [85,87]. Nitric oxide (NO), produced by epithelial NOS isoforms as discussed in Section 3.1.1, contributes further to this antimicrobial barrier through direct inhibition of microbial replication and disruption of bacterial biofilm architecture [31,32,33].

#### 3.6.3. Inflammatory Signalling and Chronic Sinusitis

Chronic inflammation of the sphenoid sinus mucosa is characterised by a shift from acute innate immune responses towards sustained adaptive immune activation, driven by T-helper cell subsets and associated cytokine environments. In the context of chronic rhinosinusitis, two principal endotypes have been identified based on the dominant inflammatory signature: a Type 2 inflammatory endotype, characterised by eosinophilic infiltration and upregulation of IL-4, IL-5, and IL-13, which predominates in allergic and aspirin-exacerbated disease; and a non-Type 2 endotype, driven by IL-17 and IFN-γ, more commonly associated with bacterial biofilm formation and neutrophilic inflammation [35,85,86,87]. The relative prevalence of these endotypes within the sphenoid sinus specifically remains incompletely characterised, but the mucosal thinness and relatively limited mucociliary reserve of the sphenoid sinus—compared with the other paranasal sinuses—may render it particularly susceptible to progression from acute to chronic inflammatory states when mucociliary clearance is impaired [86,87,88].

Persistent inflammatory signalling within the sphenoid sinus mucosa may have consequences that extend beyond the sinus itself. The elaboration of pro-inflammatory cytokines and reactive oxygen species in close proximity to the dura mater, the cavernous sinus, and the adjacent cranial nerves may contribute to perineural inflammation and subtle alterations in neurovascular function at the skull base, as discussed in Section 3.8 [18].

#### 3.6.4. Neuroendocrine and Neuropeptide Signalling

The sphenoid sinus mucosa is richly innervated by autonomic and sensory nerve fibres, whose neuropeptide mediators play important roles in the regulation of mucosal blood flow, glandular secretion, and local immune responses. Substance P and calcitonin gene-related peptide (CGRP), released from sensory C-fibres in response to noxious stimuli, promote neurogenic inflammation through mast cell degranulation, plasma extravasation, and upregulation of adhesion molecules on the endothelium of submucosal vessels [35,85]. Vasoactive intestinal peptide (VIP) and neuropeptide Y (NPY), released from parasympathetic and sympathetic fibres, respectively, exert opposing effects on glandular secretion and vascular tone, providing a neural mechanism for the fine regulation of mucosal homeostasis [35,85].

Of particular relevance to the sphenoid sinus is its proximity to the pterygopalatine ganglion—the largest parasympathetic ganglion of the head—which supplies postganglionic secretomotor fibres to the sinonasal mucosa via the vidian nerve. Pathological involvement of the pterygopalatine ganglion or the vidian canal, whether through inflammatory, neoplastic, or iatrogenic mechanisms, may therefore produce characteristic patterns of autonomic dysfunction affecting both the sphenoid sinus mucosa and the broader sinonasal region [15,73,74].

### 3.7. Anatomical Relationships with Adjacent Structures

The structures immediately adjacent to the sphenoid sinus are of critical importance for surgical planning and intraoperative orientation. They can be classified into four functional groups:

➢ Neural structures: the optic nerve (II), oculomotor nerve (III), trochlear nerve (IV), abducens nerve (VI), and the first and second branches of the trigeminal nerve (V1, V2);
➢ Vascular structures: the internal carotid artery (cavernous and paraclinoid segments), the basilar artery, and the ophthalmic artery;
➢ Glandular structures: the pituitary gland and its bony housing, the pituitary fossa (*fossa hypophysialis*);
➢ Membranous structures: the diaphragma sellae, the dura mater, and the arachnoid mater [13,14,15,18].

These structures may be more or less covered by the bony wall of the sphenoid sinus, depending on the degree of pneumatization; thinner bony coverage is associated with a progressively higher risk of intraoperative injury.

Optic nerve. The optic nerves traverse the optic canals, which extend from the orbital apex to the middle cranial fossa. In a substantial proportion of patients, the bony layer separating the optic canal from the lumen of the sphenoid sinus is very thin; in extreme cases, dehiscences in this bony wall result in direct contact between the optic nerve and the sinus mucosa, posing a major risk during endonasal surgery [14,15,16].

**Figure 5:**
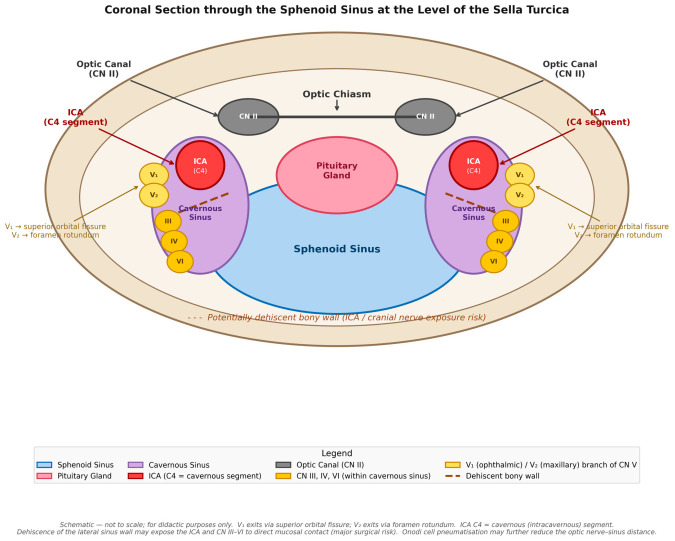
Schematic coronal section through the sphenoid sinus at the level of the sella turcica, illustrating its central position relative to adjacent neurovascular structures. The sphenoid sinus (light blue) lies inferior to the pituitary gland (pink) within the sella turcica and medial to the bilateral cavernous sinuses (purple), which contain the cavernous segment of the internal carotid arteries (C4 segment; red) and cranial nerves III, IV, V_1_, V_2_, and VI (yellow). The optic canals (grey), transmitting the optic nerves (CN II), are situated superolaterally, separated from the sphenoid sinus lumen by a bony wall of variable thickness that may be dehiscent. The ophthalmic branch (V_1_) and maxillary branch (V_2_) of the trigeminal nerve traverse the lateral wall of the cavernous sinus and exit through the superior orbital fissure and foramen rotundum, respectively. The thin bony wall separating the sphenoid sinus from the cavernous sinus (dashed brown line) may be partially or completely dehiscent in a proportion of patients, exposing the ICA and cranial nerves to direct contact with the sinus mucosa—a finding of major surgical relevance. Schematic representation—not to scale; intended for didactic purposes and not reflecting precise anatomical proportions.

Internal carotid artery. The internal carotid arteries (ICAs) pass along the lateral walls of the sphenoid sinus and represent the most critical neurovascular structures in transsphenoidal approaches (Figure 5).

Three principal anatomical conditions increase the risk of iatrogenic vascular injury:

➢ Bony dehiscence over the cavernous segment of the ICA on the lateral wall of the sinus;
➢ Protrusion of the artery into the lumen of the sinus;
➢ Reduced intercarotid (intersinus) distance, which limits the working corridor and may render simultaneous bilateral manipulation hazardous [14,15,24,81].

Preoperative assessment of these variations by computed tomography (CT) and magnetic resonance imaging (MRI) is therefore essential to safe surgical planning [1,2,19,76].

Pituitary gland and optic chiasm. The pituitary gland lies immediately superior to the sphenoid sinus and is the principal target of transsphenoidal access in pathology of the sellar region. The bony layer separating the gland from the sinus is typically thin, but its careful preservation is essential for postoperative endocrine function [17,90]. The optic chiasm, also located superior to the sinus, must be visualised on preoperative imaging—particularly in cases of suprasellar tumour extension—and incorporated into the surgical planning algorithm [77].

### 3.8. Effects of Sphenoid Sinus Pathology and Anatomical Variations on Central Nervous System Function

The intimate topographical relationships of the sphenoid sinus with critical neural and vascular structures render it a potential vector through which both anatomical variations and pathological processes may exert clinically significant effects on central nervous system function. These effects can be categorised according to the underlying mechanism: direct mechanical compression, vascular compromise, propagation of inflammatory or infectious processes, and chronic neuroinflammatory modulation [14,15,18].

Visual disturbances and optic neuropathy. The optic nerve is among the structures most vulnerable to pathological involvement originating from the sphenoid sinus. In patients with pronounced pneumatization and thin or dehiscent bony coverage over the optic canal, chronic inflammatory changes, mucocoele formation, or space-occupying lesions within the sinus may exert direct pressure on the optic nerve, producing progressive visual field defects, reduced visual acuity, or—in advanced cases—irreversible optic neuropathy [14,15,16]. The presence of Onodi cells, which position the optic nerve in immediate contact with the posterior ethmoid–sphenoid complex, further amplifies this risk. Isolated sphenoid sinusitis has been documented as a cause of sudden visual loss in the absence of obvious preceding symptoms, underscoring the clinical importance of recognising the optic nerve–sinus relationship in radiological assessment [14,16,18].

Cranial nerve neuropathies. Beyond the optic nerve, the cranial nerves coursing through or immediately adjacent to the cavernous sinus—including the oculomotor nerve (III), trochlear nerve (IV), abducens nerve (VI), and the ophthalmic and maxillary branches of the trigeminal nerve (V_1_, V_2_)—may be affected by pathological processes originating within the sphenoid sinus. Cavernous sinus syndrome, characterised by combinations of ophthalmoplegia, ptosis, facial hypoaesthesia, and proptosis, may develop as a consequence of inflammatory or infectious spread from the sphenoid sinus to the cavernous sinus, or through direct extension of a sphenoid mucocoele or neoplastic lesion [18]. Abducens nerve palsy, in particular, has been reported as a presenting sign of isolated sphenoid sinusitis, reflecting the nerve’s long intracranial course and susceptibility to compression within the cavernous sinus [14,18].

Vascular complications. The cavernous segment of the internal carotid artery, separated from the sphenoid sinus lumen by a bony wall of variable and often minimal thickness, is vulnerable to both direct mechanical injury and infectious or inflammatory involvement. Septic thrombosis of the cavernous sinus—a potentially life-threatening complication of sphenoid sinusitis—may produce cerebral ischaemia through propagation of thrombus into the cerebral venous system or through septic emboli reaching the intracranial arterial circulation [14,15,18]. In cases of extensive pneumatization with ICA dehiscence, even minor mucosal inflammatory changes may place the arterial wall in direct contact with the infectious process, representing a risk factor for mycotic aneurysm formation or arterial erosion [11,14,24].

Spread of inflammatory and infectious processes to the skull base. The sphenoid sinus occupies a central position at the skull base, and its posterior and lateral walls are in direct continuity with the dura mater and the meningeal coverings of the brainstem and posterior fossa. Bacterial or fungal sinusitis involving the sphenoid sinus may therefore spread intracranially through bony dehiscences, perineural or perivascular pathways, or direct dural erosion, giving rise to meningitis, epidural or subdural empyema, and cerebral abscess—all of which carry significant neurological morbidity and mortality [14,15,18]. Invasive fungal sinusitis, in particular, has a propensity for angioinvasion and rapid intracranial extension through the sphenoid sinus, and must be considered in immunocompromised patients presenting with cranial nerve deficits and sphenoid sinus opacification.

Neuroinflammatory and neurovascular mechanisms. Emerging evidence suggests that chronic inflammation of the sphenoid sinus may exert more subtle, sustained effects on adjacent neurovascular structures through the local elaboration of pro-inflammatory cytokines, reactive oxygen species, and vasoactive mediators. Chronic mucosal inflammation and the associated release of inflammatory mediators may influence the microvasculature of the cavernous sinus and the perineural environment of adjacent cranial nerves, potentially contributing to subclinical neuropathic changes or alterations in neurovascular coupling at the skull base [18]. Although the direct mechanistic link between chronic sphenoid sinusitis and neuroinflammatory pathology remains an area of active investigation, the anatomical contiguity of the sinus with the meningeal and neurovascular compartments provides a plausible biological basis for such interactions, warranting further clinical and experimental study.

### 3.9. Bony Landmarks

The body of the sphenoid bone (*corpus ossis sphenoidalis*), within which the sphenoid sinus is housed, contains several anatomical landmarks of particular relevance for surgical accessibility and topographic orientation:

➢ Sella turcica. A saddle-shaped depression on the superior surface of the sphenoid body that houses the pituitary gland. The shape and dimensions of the sella vary considerably between individuals, and these variations directly influence the planning of transsphenoidal access;
➢ Dorsum sellae. A bony elevation forming the posterior boundary of the sella turcica, terminating in the posterior clinoid processes;
➢ Tuberculum sellae. A small bony ridge along the anterior boundary of the sella, separating it from the planum sphenoidale and the optic chiasm above;
➢ Anterior and posterior clinoid processes. These bony projections serve as anchor points for the *diaphragma sellae* and define important neurovascular relationships in the parasellar region. The anterior clinoid processes also bound the optic canals laterally;
➢ Sphenoid (intersinus) septum. A bony partition dividing the sphenoid sinus into two—often asymmetrical—halves. Its midline position and lateral attachment points are highly variable and of considerable surgical importance, particularly when the septum terminates on the carotid sulcus or the optic canal, where surgical fracture may transmit force to the underlying neurovascular structures [14,15,16,81].

### 3.10. Anatomical Variations and Clinical Significance

The morphology of the sphenoid sinus is highly variable, and this variability carries direct clinical and surgical implications. Variations can be classified according to several criteria, including frequency (common vs. rare), morphological expression, the anatomical structure involved, and surgical relevance.

#### 3.10.1. Common Variations

The most frequently observed anatomical variations in the sphenoid sinus relate to its size and shape, the presence, number, and position of intrasinus septa, and the degree of pneumatization. Among these, the position and number of intrasinus septa are of particular practical importance in transsphenoidal surgery, since they are highly variable and may deviate from the midline in a substantial proportion of patients [11,72,75].

Intrasinus septation. The classical anatomical description specifies a single midline septum. However, in more than 60% of cases, the sphenoid septum deviates from the midline and may terminate at critical neurovascular landmarks, including the carotid canal (*canalis caroticus*), the optic canal (*canalis opticus*), or the anterior clinoid process (*processus clinoideus anterior*) [79,91]. Such terminations transform the routine intraoperative fracture of the septum into a high-risk manoeuvre, as the transmitted mechanical force may reach the underlying internal carotid artery or optic nerve. The number of septa is also variable: while a single septum is most common, two or more intrasinus partitions—including transversely oriented septa—may be present [11,72]. In addition, Onodi cells—accessory pneumatic cavities located in the posterior part of the ethmoid sinuses, in close relation to the optic nerve—may further complicate the anatomical landscape during endoscopic procedures [30,92].

Variations in pneumatization and sinus volume. Differences in the degree of pneumatization produce three principal morphological patterns:

➢ Hyperpneumatic sinus—extensive pneumatization, frequently with extensions into the greater wing, pterygoid process, or basilar part of the occipital bone;
➢ Hypopneumatic sinus—minimal pneumatization with thicker bony walls and a small air cavity;
➢ Asymmetric sinus—the right and left halves differ markedly in volume, depth, or lateral extension [14,73,93,94].

Asymmetry between the two sinus halves is observed in approximately 50% of cases [14,73,93,94]. This degree of variability mandates individualised preoperative assessment for every patient considered for transsphenoidal surgery. In particular, when the sinus extends laterally above the *foramen rotundum* or reaches the pterygoid process—corresponding to a postsellar pattern—the surgical corridor is broader, but the risk of injury to neighbouring neurovascular structures is correspondingly increased [15,16,30].

#### 3.10.2. Rare Variations

Rare anatomical variations in the sphenoid sinus encompass both congenital and acquired forms. Although less frequent than the common variations described above, each carries distinct surgical implications.

Hyperpneumatization. An extreme degree of pneumatization in which the sphenoid sinus extends into adjacent regions, including the pterygoid process, the greater wing of the sphenoid, or the basilar part of the occipital bone. This pattern is observed in approximately 8–13% of cases across published series, with reported values varying according to the classification criteria and imaging protocol applied, and overlaps morphologically with the postsellar and clival pneumatization types [14,15,69,71]. Hyperpneumatization frequently results in dehiscence of the bony layer overlying the carotid canal and the optic nerve, thereby substantially increasing the risk of iatrogenic injury during surgery.

Sinus agenesis. Complete absence of pneumatization of the sphenoid sinus, observed in less than 0.5% of the population [71,85]. In affected patients, transsphenoidal access in its standard form is not feasible, and alternative surgical corridors must be considered.

Onodi cells. Accessory pneumatic cavities located in the posterior part of the ethmoid air-cell complex, frequently in close anatomical relationship with the optic nerve and, in some cases, with the internal carotid artery. They are observed in approximately 5–15% of patients and are of considerable surgical importance, since their presence alters topographic relationships during endoscopic procedures and may lead to misidentification of the sphenoid sinus during navigation [30,92]. This range, however, substantially underestimates the prevalence reported in certain high-resolution CBCT series, where frequencies exceeding 30% have been documented. The wide variation reflects differences in imaging modality, slice thickness, and the definition applied—with some authors requiring a distinct air cell superior to the sphenoid sinus in direct contact with the optic nerve, while others accept any posterior ethmoid cell in the region [16,30,92].

The reported prevalence of these anatomical variants varies considerably across published studies, and this variability is not merely a reflection of true biological differences between populations. Several methodological factors contribute substantially to the observed heterogeneity. First, CT slice thickness exerts a direct influence on the detection of fine osseous structures: studies employing thin-slice high-resolution CT (≤1 mm) consistently report higher rates of bony dehiscences, thin bony walls, and Onodi cells compared with studies using conventional slice thicknesses of 3–5 mm, in which subtle variations may be missed or underestimated [1,2,78]. Second, differences in imaging protocols—including the plane of acquisition (axial, coronal, or multiplanar reconstruction), the use of bone versus soft-tissue windows, and the availability of three-dimensional reconstruction—affect the detectability and classification of specific variants. Third, the definitions applied to the same anatomical entity differ between authors and between classification systems: the threshold for classifying a sinus as “sellar” versus “postsellar” type, or the minimum dimensions required to identify an Onodi cell, are not universally standardised, making direct numerical comparison between studies difficult [30,42,44,71]. Fourth, sample size and case-mix introduce statistical variability, particularly for rare variants such as complete ICA dehiscence or sinus agenesis, where small series may produce wide prevalence estimates. Fifth, age composition of the study cohort is a critical confounding variable, since pneumatization continues through adolescence and may progress into adulthood; studies conducted in paediatric populations will systematically report lower rates of advanced pneumatization types than adult series [13,61]. Finally, ethnic and geographical differences in craniofacial morphology—including differences in the dimensions of the sphenoid body, the degree of skull base pneumatization, and the prevalence of specific anatomical variants—contribute genuine biological variability that cannot be attributed to methodological factors alone [30,33,42,44,85,95]. Recognition of these sources of heterogeneity is essential for the critical appraisal of the epidemiological literature on sphenoid sinus morphology and for the appropriate contextualisation of prevalence data within specific clinical and population settings (Table 1).

Accessory ostia. Additional openings between the sphenoid sinus and the nasal cavity, supplementary to the principal sphenoid ostium. Although uncommon, accessory ostia can complicate both diagnostic interpretation and surgical orientation, particularly in revision surgery [80,81].

These rare variations, while individually uncommon, are of paramount importance for surgical planning, particularly in endoscopic approaches. Their recognition requires careful preoperative imaging analysis using CT and MRI to avoid serious complications, including cerebrospinal fluid (CSF) leakage and injury to adjacent neurovascular structures [1,2,19].

### 3.11. Radiological and Surgical Classifications

A number of radiological and surgical classification systems have been developed to systematise the anatomical variations in the sphenoid sinus and to support preoperative decision-making. These classifications are based on the assessment of sinus morphology and adjacent neurovascular structures using computed tomography (CT) and magnetic resonance imaging (MRI), and they differ according to the anatomical parameter they emphasise [14,69,70,71].

#### 3.11.1. Radiological Classifications

Two of the most widely cited radiological classifications are the Hamberger classification and the Vaezi classification. The Hamberger classification categorises the sphenoid sinus according to the anteroposterior extent of pneumatization relative to two bony reference points: the tuberculum sellae (T) and the dorsum sellae (D). Four principal types are defined—conchal, presellar, sellar, and postsellar—reflecting progressively greater posterior extension of pneumatization [69,71]. The Vaezi classification addresses a complementary anatomical dimension: the lateral development of the sinus into the pterygoid, lateral, and clival recesses, categorised on a scale of Type 0 (absent recess) through Type 3 (fully developed recess extending into the pterygoid root or greater wing). Both systems are derived from CT-based morphometric analysis and are widely applied in radiological and surgical planning contexts [14,69,70,71].

A complementary classification addresses the morphology of the intrasinus septation, which is of particular surgical relevance:

➢ Type I—a single septum running strictly along the midline of the sinus, dividing it into two relatively symmetrical halves;
➢ Type II—a single septum deviated from the midline, frequently attached at or near the lateral wall of the sinus in close relation to the carotid canal;
➢ Type III—multiple septa (two or more), dividing the sinus into more than two compartments with additional bony partitions;
➢ Type IV—accessory septa of smaller size, frequently incomplete, forming additional small cells or recesses within the main sinus cavity;
➢ Type V—atypical septum of irregular shape and orientation, often deviated and combined with other intrasinus structures, complicating both radiological interpretation and surgical management [11,72,75,79].

This classification is of direct surgical relevance: deviated or atypical septa (Types II and V in particular) may direct intraoperative fracture forces towards the internal carotid artery or other critical neurovascular structures. It should be noted that this five-type septation classification represents a descriptive radiological framework based on the morphometric and anatomical data synthesised in the present review, informed by the existing literature on intrasinus septal patterns [11,72,75,79]. It has not been formally validated in a prospective clinical cohort, nor has it been adopted as a standardised system in the published literature; it is presented here as a structured descriptive tool to facilitate the systematic reporting and surgical appraisal of intrasinus septation patterns. Clinicians should interpret it in conjunction with established classification frameworks and individualised preoperative imaging.

#### 3.11.2. Surgical Classifications

Surgical classifications focus less on the intrinsic morphology of the sinus and more on the relationships between the sinus and adjacent structures critical for transsphenoidal access.

The Eichenberg classification was developed in the context of extended endoscopic endonasal approaches and categorises the sphenoid sinus according to the depth and lateral extent of pneumatization, with specific attention to the development of the pterygoid and clival recesses. It provides a framework for selecting appropriate surgical instruments and planning access corridors for complex sellar, parasellar, and skull base pathology, including lesions of the cavernous sinus, the petroclival region, and the upper clivus. The classification assists the surgeon in anticipating the degree of bony work required and in identifying the anatomical boundaries of the operative corridor prior to the procedure [17,90].

The Kassam classification was developed specifically for transsphenoidal skull base surgery and focuses on the topographic relationships between the sphenoid sinus and three anatomical structures of critical surgical importance: the sella turcica, the internal carotid arteries, and the optic nerves. By characterising the degree of sinus pneumatization in relation to these structures, the Kassam classification stratifies the surgical complexity of transsphenoidal access and assists in optimising intraoperative visualisation, defining the limits of safe dissection, and minimising the risk of neurovascular injury during extended approaches [17,90]. It is particularly applicable in the context of pituitary adenomas with significant suprasellar or parasellar extension, where the spatial relationships between the tumour, the sinus, and the adjacent neurovascular structures determine the surgical strategy (Table 2).

The choice of classification depends on the clinical context: radiological classifications are particularly useful for preoperative imaging-based assessment, whereas surgical classifications guide instrument selection and operative strategy.

### 3.12. Radiological Assessment of Morphological Variations

Computed tomography (CT) and magnetic resonance imaging (MRI) are the two principal imaging modalities for preoperative assessment of the sphenoid sinus, each with distinct indications and limitations [1,2,78].

#### 3.12.1. Computed Tomography (CT)

Examination protocols. Computed tomography is the foundational method for evaluation of the bony anatomy of the sphenoid sinus and its surrounding structures. It is widely regarded as the gold standard for visualisation of bone, and therefore represents the imaging modality of choice for identifying variations in pneumatization, intrasinus septa, bony dehiscences, and other osseous defects [1,2,78].

The standard imaging protocol for the sphenoid sinus and parasellar region includes a series of axial sections of 0.5–1.5 mm slice thickness, enabling the reconstruction of three-dimensional models with high spatial fidelity [19,20]. The use of high-resolution CT (HRCT) is preferred, as it provides superior detail of the bony architecture. Multiplanar coronal and sagittal reconstructions are essential for visualising the spatial relationships between the sphenoid sinus and adjacent structures, including the internal carotid arteries, the optic chiasm, and the cranial nerves traversing the cavernous sinus [1,2,76].

Limitations. CT provides insufficient resolution of the soft-tissue structures within the parasellar region, including the mucosa of the sphenoid sinus, the pituitary gland, and the contents of the cavernous sinus. In addition, CT exposes the patient to ionising radiation, which constitutes a relative limitation for repeated imaging, particularly in younger patients and in long-term follow-up scenarios [2,14,15].

#### 3.12.2. Magnetic Resonance Imaging (MRI)

Examination protocols. Magnetic resonance imaging is widely regarded as the gold standard for visualisation of soft tissues, including the pituitary gland, the surrounding parasellar structures, and the contents of the cavernous sinus [1,2]. The standard MRI protocol for the sellar and parasellar region includes the following sequences:

➢ T1-weighted sequences, performed both before and after intravenous administration of gadolinium-based contrast, providing high anatomical detail and enhanced visualisation of vascular and pathological structures;
➢ T2-weighted sequences, useful for the characterisation of cystic lesions, fluid-containing cavities, and the assessment of mucosal inflammation;
➢ Fluid-Attenuated Inversion Recovery (FLAIR), for the assessment of perilesional oedema and inflammatory processes;
➢ Dynamic contrast-enhanced sequences, used to evaluate pituitary gland perfusion and to differentiate normal gland tissue from microadenomas [1,2].

Thin-section (≤3 mm) acquisitions are typically used for the sellar region, with coronal and sagittal planes providing complementary information on the spatial relationships of the pituitary gland and its surrounding structures. Diagnostic capabilities. MRI provides sensitive and specific assessment of the soft-tissue components of the parasellar region. Its principal advantages over CT include:

➢ Detailed soft-tissue visualisation of the pituitary gland, allowing diagnosis of microadenomas, macroadenomas, and other intrasellar and suprasellar pathologies;
➢ Excellent depiction of neurovascular structures, including the cavernous sinus, the optic nerves, and the internal carotid arteries—particularly important for assessing tumour invasion of the cavernous sinus or contact with the optic apparatus;
➢ Absence of ionising radiation, making MRI the preferred modality for repeated imaging, paediatric patients, and longitudinal follow-up of dynamic changes in the parasellar region [1,2,3,4,5,6,7,8,9,10,18,28,33,57].

Limitations. MRI does not provide the level of bony detail required for the visualisation of intrasinus septa, dehiscences, or fine osseous architecture; for these assessments, CT remains essential. Furthermore, MRI examinations require considerably longer acquisition times than CT and are sensitive to motion artefact, which may limit their utility in emergency surgical contexts or in patients unable to remain still. Standard contraindications—including non-MRI-compatible implants, certain cardiac devices, and severe claustrophobia—further restrict the applicability of MRI in selected cases [2,14].

#### 3.12.3. Comparative Analysis: CT vs. MRI

CT and MRI should be regarded as complementary rather than competing modalities in the radiological assessment of the sphenoid sinus and parasellar region. CT remains the method of choice for the evaluation of bony anatomy and the detection of fine osseous variations, while MRI is indispensable for the characterisation of soft-tissue pathology, the pituitary gland, and the contents of the cavernous sinus. The principal differences between the two modalities are summarised in Table 3.

In clinical practice, CT and MRI are most often used in combination, with the sequence and emphasis determined by the clinical context [19,20,76,78].

#### 3.12.4. Clinical Algorithms for Imaging Selection

A typical decision algorithm proceeds as follows:

➢ Initial assessment. Computed tomography is generally performed first to evaluate the bony anatomy of the sphenoid sinus and to identify osseous variations relevant to surgical risk, including intrasinus septa, dehiscences over the carotid canal and optic nerve, and the morphology of pneumatization [1,2].
➢ Surgical planning. Once osseous anatomy is characterised, MRI is acquired for detailed evaluation of soft-tissue structures, including the pituitary gland, the cavernous sinus, and any associated pathological process. The combination of CT and MRI—frequently co-registered into a single fused volume—provides a comprehensive three-dimensional view that supports precise planning of the operative corridor and instrumentation strategy [19,20,76,78].
➢ Postoperative monitoring. MRI is the preferred modality for postoperative follow-up, owing to the absence of ionising radiation and its sensitivity to subtle soft-tissue changes such as residual tumour, recurrence, or post-surgical inflammation [1,2,19,20,76,78].

The selection of additional or alternative imaging modalities—including angiographic techniques (CT angiography, MR angiography) for vascular assessment—is determined by the specific clinical scenario, the suspected pathology, and institutional protocols.

### 3.13. Emerging Technologies in Surgical Planning

Beyond conventional CT and MRI, recent advances in immersive digital technologies have begun to influence the preoperative planning and intraoperative execution of transsphenoidal procedures. Among these, virtual reality (VR) and augmented reality (AR) have shown particular promise in addressing the challenges posed by the complex morphology of the sphenoid sinus and its surrounding neurovascular structures [20,87,88,96].

#### 3.13.1. Virtual Reality

Virtual reality offers an immersive preoperative environment in which surgeons can interact with three-dimensional reconstructions of patient-specific anatomy. Through head-mounted displays, the operator is placed within a virtual representation of the surgical field, enabling a more intuitive appreciation of spatial relationships and anatomical variations than conventional two-dimensional imaging can provide [20,87,88,96]. The principal applications of VR in transsphenoidal surgery include:

➢ Patient-specific simulations. VR platforms can reproduce the individual anatomy of a given patient with high fidelity, allowing the surgeon to rehearse the planned procedure virtually and to refine the operative strategy before entering the operating room. This is particularly valuable in cases of complex anatomical variations, such as extensive pneumatization, dehiscences over the internal carotid canal, or atypical septa [18,87,88];
➢ Visualisation of anatomical variations. VR enables three-dimensional visualisation of complex morphological variations, providing a clearer understanding of the patient’s individual anatomy and the relationships among the internal carotid artery, the optic nerve, and the cavernous sinus [76,87,88,90,91,92,94,95,96];
➢ Surgical workflow simulation. Surgeons can simulate the entire operative workflow, identify potential difficulties at each step, and develop strategies to address them. This contributes to improved preoperative preparation and may reduce the risk of intraoperative complications [95,96];
➢ Training and skill acquisition. Surgical residents and less experienced practitioners can develop technical and cognitive skills in a risk-free virtual environment, repeating challenging procedures multiple times before transitioning to real patient care [86,95].

Despite this considerable promise, the clinical implementation of VR in transsphenoidal surgery remains at an early stage. The majority of published evidence derives from feasibility studies, single-centre series, and educational validation studies rather than from prospective randomised trials assessing patient outcomes. Key limitations include the high cost of VR hardware and software platforms, the technical expertise required for anatomical model generation from CT/MRI datasets, and the time investment associated with producing patient-specific virtual reconstructions in routine clinical practice. The quality of VR models is directly dependent on the resolution and quality of the underlying imaging data, and artefacts in CT or MRI acquisitions may compromise the fidelity of the virtual representation. Furthermore, VR environments currently lack haptic feedback—the tactile sensation of tissue resistance during dissection—which limits their value as simulators of the full surgical experience. Standardisation of VR training curricula and objective metrics for competency assessment remain to be established [97,98,99,100].

#### 3.13.2. Augmented Reality

Augmented reality differs from virtual reality in that it overlays digital information onto the real surgical field, rather than replacing it entirely. AR devices project three-dimensional reconstructions derived from CT or MRI directly onto the patient or onto a head-mounted display, providing the surgeon with a real-time view of the underlying anatomical structures during the operation [20,87,95,96]. The principal applications of AR in transsphenoidal surgery include:

➢ Intraoperative navigation. AR enables real-time visualisation of critical structures—including the sphenoid sinus, the optic nerves, the internal carotid arteries, and other neurovascular landmarks—directly within the operative field. This continuous spatial referencing may substantially reduce the risk of inadvertent injury during instrument manipulation;
➢ Enhanced operative precision. By superimposing preoperative imaging data onto the live surgical scene, AR allows more accurate identification of pathological lesions and assists the surgeon in adjusting the approach to ensure complete resection while preserving important neurological functions;
➢ Support for intraoperative decision-making. AR can provide additional contextual information—such as the position of critical structures, surgical margins, or trajectory planning—supporting rapid and informed decision-making during the procedure, particularly in anatomically challenging cases [18,34,35,76,83,84,85,86,87,88,89,95,96].

The clinical adoption of AR in transsphenoidal surgery faces several challenges that currently limit its routine implementation. Registration accuracy—the precision with which the digital overlay is aligned to the real anatomy—remains a critical technical concern, as even small registration errors may misdirect surgical manoeuvres in the vicinity of the ICA or optic nerve. Soft-tissue deformation during surgery, brain shift following tumour resection, and endoscope movement all introduce dynamic registration inaccuracies that current AR systems do not fully compensate for in real time. The available evidence base consists predominantly of proof-of-concept studies and early clinical series; prospective comparative data demonstrating measurable improvements in surgical outcomes—including complication rates, extent of resection, or operative time—are lacking [97,98,99]. Additionally, the integration of AR with existing endoscopic and neuronavigation platforms requires bespoke software solutions that are not yet universally available, and the ergonomic demands of head-mounted AR displays during prolonged procedures remain a practical concern. Regulatory clearance for intraoperative AR guidance systems varies considerably across jurisdictions, adding a further barrier to widespread adoption [97,99,100].

VR, AR, mixed reality, neuronavigation, and AI-assisted segmentation collectively represent a new generation of digital tools for surgical planning and intraoperative guidance. Their broader clinical adoption depends on continued advances in hardware ergonomics, software integration with intraoperative navigation systems, regulatory validation, and the accumulation of prospective evidence demonstrating measurable improvements in patient-centred surgical outcomes [97,98,99,100].

#### 3.13.3. Neuronavigation and Mixed Reality

Image-guided neuronavigation represents an established and widely validated technology in contemporary skull base surgery, and its integration with transsphenoidal approaches has been extensively documented [97,98,99]. Frameless stereotactic neuronavigation systems register preoperative CT and MRI datasets to the patient’s intraoperative position using surface or fiducial-based registration, allowing the surgeon to track the position of instruments in real time relative to the preoperatively defined anatomy. In transsphenoidal surgery, neuronavigation is of particular value in cases of limited or absent pneumatization, atypical septal configurations, or prior surgical intervention, where conventional anatomical landmarks may be absent or distorted [97,99]. Reported accuracy of contemporary systems ranges from 1 to 2 mm in clinical use, though registration error may increase in the context of brain shift or soft-tissue deformation during resection.

Mixed reality (MR) represents an emerging convergence of virtual and augmented reality technologies, in which holographic three-dimensional reconstructions derived from CT and MRI are rendered in the surgeon’s field of view and anchored to real-world objects—including the patient’s anatomy—using spatial computing platforms. Unlike conventional AR, mixed reality holograms can be interactively manipulated, scaled, and repositioned in real time, enabling dynamic surgical rehearsal and intraoperative reference within the same platform [98,100]. Early clinical reports have demonstrated the feasibility of mixed reality guidance in endoscopic endonasal skull base procedures, with surgeons reporting improved spatial orientation and reduced cognitive load during complex anatomical dissection [97,99]. Standardised validation protocols and prospective outcome data remain limited, and the integration of mixed reality with existing neuronavigation infrastructure represents an active area of technical development.

#### 3.13.4. Artificial Intelligence, Machine Learning, and Automated Segmentation

The application of artificial intelligence (AI) and machine learning (ML) to the radiological assessment of the sphenoid sinus and skull base anatomy is rapidly advancing, with demonstrated potential to improve both the efficiency and accuracy of preoperative planning [15,88]. Deep learning algorithms—particularly convolutional neural networks (CNNs) trained on large annotated CT and MRI datasets—have shown strong performance in the automated segmentation of paranasal sinus anatomy, achieving accuracy comparable to or exceeding that of experienced radiologists in the delineation of critical structures including the sphenoid sinus walls, the internal carotid artery, the optic nerve, and the cavernous sinus [15,88].

Automated segmentation offers several advantages over manual delineation: it substantially reduces the time required to generate three-dimensional anatomical reconstructions, eliminates inter-observer variability in structure identification, and enables the systematic extraction of morphometric data—including sinus volume, wall thickness, and intercarotid distance—from large patient cohorts in a reproducible and scalable manner [15,88]. These capabilities are directly relevant to the epidemiological aims of the present review, as AI-assisted morphometry could in principle generate the large, standardised, population-stratified datasets that are currently lacking in the sphenoid sinus literature.

Beyond segmentation, AI applications in skull base surgery include computer vision-based instrument tracking, predictive modelling of surgical difficulty based on preoperative imaging features, and automated risk stratification for neurovascular complications. Convolutional neural networks have also been applied to the classification of pneumatization types and the detection of Onodi cells and bony dehiscences from CT scans, with results suggesting that automated classification can approach the diagnostic accuracy of specialist radiologists [15,88]. The integration of AI-derived anatomical models with VR and AR platforms represents a further frontier, enabling the generation of fully automated, patient-specific immersive surgical rehearsal environments from standard preoperative imaging without manual segmentation [97,98,99,100].

Despite this promise, several limitations of current AI applications warrant acknowledgement. Training datasets are frequently small, single-centre, and ethnically homogeneous, limiting the generalisability of published models. Ground-truth segmentation labels used for training are themselves subject to inter-observer variability, potentially propagating systematic errors into the trained model. Regulatory frameworks for the clinical deployment of AI-based medical devices remain in development in most jurisdictions, and prospective validation of AI-assisted surgical planning tools in terms of patient-centred outcomes—including complication rates, operative time, and completeness of resection—has yet to be established [15,88].

## 4. Discussion

The present narrative review has synthesised the contemporary literature on the morphological and epidemiological characteristics of the sphenoid sinus, with emphasis on its developmental anatomy, classification systems, anatomical variations, radiological assessment, and the emerging role of immersive technologies in surgical planning. The accumulated evidence reveals an anatomically dynamic structure whose variability is simultaneously a defining feature and a principal clinical challenge. Several interrelated themes emerge from this synthesis.

Morphological variability is the rule, not the exception. Across virtually every parameter examined in the literature—pneumatization pattern, intrasinus septation, bony wall thickness, neurovascular relationships, and overall sinus volume—the sphenoid sinus exhibits pronounced inter-individual variability [30,71,72,76]. The prevalence of the principal pneumatization types fluctuates considerably across studies: the conchal type ranges from approximately 1–3%, the presellar type around 14%, the sellar type approximately 75%, and the postsellar (hyperpneumatic) type 8–13%, with the clival type representing the maximal pneumatization pattern found exclusively in adults [69,71]. Population-based investigations from Pakistani, Romanian, Indian, Iranian, Turkish, and Bulgarian cohorts have repeatedly demonstrated that the distribution of these morphotypes is not constant across populations; certain pneumatization patterns appear to be more prevalent in particular demographic groups, although the methodological heterogeneity between studies precludes definitive quantitative comparison [30,33,42,44,85,95].

The sources of this heterogeneity merit explicit discussion. Reported prevalence values for the principal pneumatization types, Onodi cells, intrasinus septal patterns, and neurovascular dehiscences vary substantially between studies, and this variation reflects both genuine biological differences and methodological inconsistencies. CT slice thickness is among the most important technical determinants: high-resolution thin-slice protocols (≤1 mm) yield substantially higher detection rates for fine osseous variants than conventional protocols [30,33,42,44]. Differences in classification criteria—for example, the threshold distinguishing the sellar from the postsellar type, or the minimum dimensions required to identify an Onodi cell—further compound cross-study comparability. Age composition, sample size, and the use of clinical versus population-based cohorts introduce additional variability. These considerations do not diminish the clinical relevance of the reported data, but they underscore the importance of interpreting individual prevalence estimates within their specific methodological context rather than as universal reference values [30,33,42,44,71,85,95].

The surgical implications of this variability are direct and measurable. The conchal and presellar types, characterised by limited pneumatization, substantially increase operative difficulty during transsphenoidal access—requiring extensive bony drilling, prolonging operative time, and reducing the availability of intraoperative anatomical landmarks [72,76,96]. Conversely, the sellar type provides optimal working conditions for standard endoscopic endonasal approaches to the pituitary gland, explaining its central role in the contemporary surgical literature [69,71,72]. The postsellar and clival types, while facilitating wide surgical exposure in extended skull base approaches, paradoxically increase the risk of neurovascular injury through progressive thinning of the bony coverage over the cavernous ICA and optic nerve [18,71,72,96]. These observations have been corroborated by independent cohort studies, including those of Hamid et al. [72] and Gopalakrishnan et al. [55], who demonstrated a significant association between pneumatization pattern and the complexity of sellar exposure and risk of residual disease in pituitary surgery.

The cohort reported by Bechev et al. (2024) [24], based on MRI assessment of 112 patients, illustrates this point on a parameter of immediate surgical relevance—the intercarotid distance at the cavernous segment of the internal carotid artery. Even within a single national population, the observed range of values is wide, and the variability cannot be predicted from external clinical features alone. Embryologically, this variability is rooted in the complex, stepwise process of pneumatization, in which the timing, direction, and ultimate extent of bone marrow involution and air-cell expansion differ between individuals—and even between the two halves of the same sinus, which mature asynchronously in approximately half of the population [37,71]. These observations carry a single practical implication: standard anatomical atlases, however authoritative, cannot substitute for individualised preoperative imaging analysis [50,51]. The notion of a “typical” sphenoid sinus is a didactic convenience rather than a clinical reality.

Imaging integration drives the modern approach. The complementary nature of CT and MRI for the assessment of the sphenoid sinus has long been recognised [19,20,76], but the increasingly routine integration of the two modalities into co-registered three-dimensional reconstructions represents a substantial advance over either modality alone [19,20,63]. CT provides definitive information on the osseous variations critical for surgical risk—intrasinus septa, bony dehiscences over the carotid canal and optic nerve, and the detailed morphology of pneumatization [10,71,76]. MRI, in turn, characterises the soft-tissue environment, the pituitary gland, and any pathological process that motivates intervention [23,24,77]. Importantly, the addition of angiographic techniques (CT angiography and MR angiography) extends this assessment to the cerebral vasculature, which is essential when ICA tortuosity or aneurysmal disease is suspected [24,76].

The synthesis of CT, MRI, and, where indicated, angiographic data into a unified preoperative dataset represents the contemporary standard of care [19,20,76,78]. AI-assisted segmentation has the potential to reduce inter-observer variability and accelerate three-dimensional reconstruction, though prospective clinical validation remains limited [15,88].

Emerging technologies extend, rather than replace, conventional imaging. The role of virtual and augmented reality in transsphenoidal surgical planning is rapidly expanding, and the available literature describes a growing range of clinical and educational applications [97,98,99,100]. VR enables patient-specific rehearsal of complex procedures in a risk-free environment, supporting both case-specific planning and the acquisition of surgical skills by residents [100]. AR offers real-time overlay of anatomical information onto the operative field, providing continuous spatial referencing during the procedure itself [97,98,99]. These technologies do not supersede conventional CT and MRI; rather, they exploit the data acquired by these modalities, transforming them into immersive, interactive representations that more closely align with the cognitive demands of complex surgery [97,99,100].

Nonetheless, the evidence base for the clinical impact of VR and AR remains in active development [97,99]. Most published studies to date have focused on feasibility, technological validation, and surgeon acceptability, while prospective comparative studies assessing patient-centred outcomes—operative time, complication rates, completeness of resection, and length of hospital stay—remain relatively few [98,99]. Practical considerations also influence the rate of adoption: VR and AR systems remain costly, require specialised training for effective use, and depend on robust software–hardware integration that may not be universally available, particularly in lower-resource settings [97,100]. The trajectory of these technologies over the coming decade is likely to be shaped by both technological maturation and convergence with adjacent fields such as surgical robotics and AI-assisted intraoperative decision support [15,98,100].

A conceptual summary of the principal findings of this review—integrating the developmental, morphological, radiological, and technological dimensions of sphenoid sinus assessment—is presented in Table 4.

Strengths of the Present Review. The present synthesis offers several strengths. It draws on a broad and contemporary body of literature, covering the developmental, anatomical, radiological, and technological dimensions of sphenoid sinus morphology within a single integrative framework [13,30,50,51,71,72]. It incorporates data from multiple population cohorts, providing a geographically diverse perspective rather than a single-region view [30,33,42,44,85,95]. It also addresses both established imaging modalities and the rapidly evolving immersive technologies, situating the latter within the broader context of contemporary surgical practice [97,98,99,100].

Limitations of the Study. Several limitations of the present review must be explicitly acknowledged. First, as a narrative rather than systematic review, the present synthesis does not employ formal risk-of-bias assessment, quantitative meta-analysis, or pre-registered selection criteria; the reader should regard the conclusions as an expert synthesis rather than as the output of a fully reproducible methodology. Despite the systematic approach to literature identification—conducted across four major databases with a structured search strategy -the review does not claim to provide an exhaustive analysis of all available sources on the morphology of the sphenoid sinus; the selection and interpretation of the included literature necessarily reflect the authors’ clinical and academic perspective.

Second, the findings of the review may be subject to limitations introduced by the selected databases and search strategy. Restriction to English-language publications may have excluded relevant work published in other languages—a limitation of particular relevance to a topic on which substantial regional research has been published in Slavic, Romance, and Asian linguistic contexts. The time range of the search (January 2000—March 2026), while broad, excludes older anatomical literature that may contain foundational morphometric data not yet digitised or indexed in contemporary databases.

Third, the process of conceptual synthesis inherently involves subjective judgement in the selection, weighting, and interpretation of evidence. Although the literature search and eligibility assessment were conducted systematically and verified by a second author, the thematic organisation of findings and the formulation of clinical implications represent interpretive decisions that may differ between reviewers. This is an inherent characteristic of the narrative review methodology and should be considered when applying the conclusions of this synthesis to specific clinical contexts.

Fourth, the epidemiological data extracted from individual cohort studies are subject to substantial heterogeneity in imaging protocols, measurement techniques, and population characteristics, which limits direct cross-study comparison and precludes pooled quantitative estimates [30,42,44,85]. Finally, the literature on immersive technologies is evolving rapidly; further key publications may emerge in the interval between the present search update and publication, and the possibility of publication bias—particularly with respect to negative or null findings concerning VR and AR—should be considered when interpreting the current body of evidence [97,99].

Implications and future directions. The findings of this review have several implications for both clinical practice and future research. For surgical practice, the emphasis on individualised preoperative assessment—using fused CT/MRI datasets and, where available, VR-based rehearsal—should be regarded as the contemporary standard of care [19,20,63,76], particularly in cases involving extensive pneumatization, atypical septation, or known neurovascular variations [18,72,96]. For surgical education, the integration of immersive simulation into residency curricula offers a means of acquiring three-dimensional anatomical reasoning that complements traditional cadaveric and observational training [100].

For research, several priorities emerge:

➢ The development of standardised morphometric protocols that enable meaningful cross-population comparison and the construction of robust normative databases [24,30,71];
➢ Prospective, comparative studies of VR- and AR-assisted surgery versus conventional planning, employing patient-centred outcome measures including operative time, complication incidence, completeness of resection, and quality of life [97,98,99];
➢ Further characterisation of the relationship between specific anatomical variations and intraoperative complication rates, particularly in relation to ICA dehiscence, atypical septal terminations, and Onodi cell variants [9,18,72,96];
➢ The integration of artificial-intelligence-based segmentation and automated risk stratification into preoperative imaging workflows [15,88];
➢ International collaborative datasets that include underrepresented regions and demographic groups, supporting a more globally representative understanding of sphenoid sinus morphology [30,44,85,95].

The continued accumulation of high-quality, population-stratified data—together with the maturation of immersive technologies and their integration into established surgical workflows—will be central to refining our understanding of sphenoid sinus morphology and to translating this knowledge into safer, more effective transsphenoidal surgery [19,20,71,72,97,99].

## 5. Conclusions

The sphenoid sinus represents one of the most anatomically variable pneumatic structures of the human skull base. Its central position at the cranial base and intimate relationships with the pituitary gland, the optic apparatus, the internal carotid arteries, and cranial nerves III–VI render it both a critical anatomical landmark and a surgical corridor of considerable complexity. The morphological characteristics of the sinus—pneumatization pattern, intrasinus septation, bony wall thickness, and the configuration of adjacent neurovascular structures—exhibit substantial inter-individual variability across all population cohorts studied to date.

This variability has direct and unavoidable consequences for surgical practice. Each transsphenoidal procedure requires individualised preoperative assessment, with no anatomical template applicable to all patients. The integration of CT and MRI, ideally co-registered into a unified three-dimensional dataset, provides the contemporary foundation for this assessment: CT delineates the osseous architecture critical to surgical risk, while MRI characterises the soft-tissue environment and the pathological processes that motivate intervention. Angiographic techniques further support this assessment when vascular anatomy or pathology is of particular concern. Emerging immersive technologies—virtual reality and augmented reality—are progressively extending these capabilities by transforming static imaging datasets into interactive, patient-specific surgical rehearsals and real-time intraoperative overlays. Although their clinical impact requires further prospective validation, these tools are likely to become integral components of skull base surgery within the coming decade, particularly when combined with advances in surgical robotics and artificial-intelligence-assisted decision support.

For clinical practice, the principal message of the present synthesis is that detailed morphological knowledge of the sphenoid sinus is not an academic abstraction, but plays an important role in surgical strategy, instrument selection, and risk mitigation. For research, the priority lies in the development of standardised morphometric protocols, the accumulation of population-stratified data, and rigorous evaluation of the patient-centred outcomes of emerging technologies. The continued convergence of anatomical knowledge, advanced imaging, and immersive digital tools is expected to translate, in time, into safer and more effective transsphenoidal interventions.

## Figures and Tables

**Table 1:** Comparative prevalence of major sphenoid sinus anatomical variants across population-based cohort studies, stratified by geographical region, sample size, and imaging modality.

Study/Author	Country/Region	n	Modality	Conchal	Presellar	Sellar	Postsellar	ICA Protrusion	ON Protrusion	Onodi Cells	Ref.
A. Pneumatization Types											
Hamberger	Multiple/Historical	Large series	Rad/CT	~3%	~14%	~75%	~8%	—	—	—	[69]
Açar et al. 2024	Turkey	300	CT	0.7%	6.6%	30%	62.7%	—	—	—	[30]
Dogan et al. 2023	Turkey	209	CT	1.4%	8.1%	79.9%	10.5%	—	—	—	[71]
Sagar et al. 2023	India	114	CT	5.2%	26.3%	68.4% *	—	—	—	—	[75]
Tavakoli et al. 2023	Iran	Variable	CBCT	—	7%	36%	56%	31%	21%	—	[42]
Aijaz et al. 2023	Pakistan	300	CT	Rare	Minority	Majority	Present	Present	Present	—	[85]
Rahmati et al. 2016	Iran	Variable	CBCT	Present	Present	Majority	Present	—	—	—	[44]
Hamid et al. 2008	Egypt	Variable	CT/MRI	—	—	Majority	—	Present	Present	—	[72]
B. Neurovascular Variants (ICA and Optic Nerve)											
Jaworek-Troć et al. 2021	Poland	Variable	CT	—	—	—	—	Present	—	—	[9]
Sirikci et al. 2000	Turkey	Variable	CT	—	—	—	—	Present	Present	—	[11]
Kanotra et al. 2023	India	Variable	CT	—	—	—	—	—	Variable	—	[33]
Gruszka et al. 2022	Poland/Cyprus	210	CBCT	—	—	—	—	—	—	Variable	[16]
Bechev et al. 2024	Bulgaria	112	MRI	—	—	—	—	Variable	—	—	[24]
C. Onodi Cells											
Hassan et al. 2024	Variable	Variable	CT	—	—	—	—	—	—	~5–15%	[92]
Gruszka et al. 2022	Poland/Cyprus	210	CBCT	—	—	—	—	—	—	>15%	[16]

Abbreviations: CT = computed tomography; CBCT = cone-beam CT; MRI = magnetic resonance imaging; ICA = internal carotid artery; ON = optic nerve; Ref. = reference. * Sellar type as reported by Sagar et al. includes both sellar and postsellar subtypes under a combined category. Bechev et al. [24] report morphometric data (intercarotid distance) rather than dichotomous protrusion/dehiscence rates. Values marked ‘Present’ indicate the variant was documented but exact prevalence was not extractable from the available abstract or citation. Values marked ‘Variable’ indicate substantial inter-study heterogeneity as discussed in Section 3.10. Note: Inter-study variability reflects genuine population differences as well as methodological factors including CT slice thickness, imaging protocol, classification criteria, and age composition of the study cohort. Data should be interpreted within their specific methodological context (see Section 3.10 for detailed discussion).

**Table 2:** Comparison of principal classification systems for sphenoid sinus morphology and surgical planning.

Classification	Author/Year	Basis	Types/Categories	Clinical Relevance	Limitations
Hamberger	Hamberger et al., 1961 [69]	Anteroposterior extent of pneumatization relative to tuberculum sellae (T) and dorsum sellae (D)	Conchal, presellar, sellar, postsellar (hyperpneumatic)	High—directly predicts transsphenoidal working space and landmark availability	Does not account for lateral pneumatization or recess development
Güldner/Wang	Güldner et al.; Wang et al. [5]	Lateral and inferior extension of pneumatization beyond the sphenoid body	Types based on pterygoid and clival extension	Moderate—relevant for extended endoscopic approaches	Less widely adopted; limited validation data
Vaezi	Vaezi et al. [71]	Development of lateral and pterygoid recesses	Types 0–3 (absent to fully developed lateral recess)	High—determines access to cavernous sinus, foramen rotundum, and vidian canal	Requires high-resolution CT; less useful for standard sellar approaches
Bilgir	Bilgir et al. [5]	Combined morphometric and pneumatization assessment	Multiple subtypes	Moderate—useful for population-based morphometric studies	Limited surgical applicability compared to Hamberger and Vaezi
Septation classification (Types I–V)	Radiological—Section 3.11	Number, position, and orientation of intrasinus septa	Type I: single midline; Type II: deviated; Type III: multiple; Type IV: transverse; Type V: complex	Very high—septal termination on carotid sulcus or optic canal defines intraoperative fracture risk	Not universally standardised; validation data limited
Eichenberg	Eichenberg et al. [17,90]	Depth and extent of pneumatization adapted for extended endoscopic endonasal approaches	Grades based on depth of sinus extension	High—guides instrument selection and corridor planning for complex sellar and parasellar pathology	Primarily applicable to extended approaches; less relevant for standard transsphenoidal access
Kassam	Kassam et al. [17,90]	Topographic relationships of sinus to sella turcica, ICA, and optic nerves	Subtypes based on neurovascular proximity	Very high—specifically designed to optimise intraoperative visualisation and minimise neurovascular risk in extended skull base approaches	Requires detailed preoperative CT/MRI co-registration; complex to apply without dedicated imaging workstation

**Table 3:** Comparative analysis of computed tomography (CT) and magnetic resonance imaging (MRI) for assessment of the sphenoid sinus and parasellar region.

Parameter	CT	MRI
Primary diagnostic strength	Bony anatomy	Soft-tissue contrast
Typical slice thickness	0.5–1.5 mm	≤3 mm
Visualisation of intrasinus septa	Excellent	Limited
Visualisation of bony dehiscences	Excellent	Poor
Identification of Onodi cells	Excellent	Moderate
Assessment of pituitary gland	Limited	Excellent
Assessment of cavernous sinus content (CN III–VI, ICA)	Moderate	Excellent
Detection of soft-tissue tumour invasion	Poor	Excellent
Ionising radiation	Yes	No
Acquisition time	Minutes	15–45 min (protocol-dependent; dedicated pituitary protocols typically 15–20 min)
Contraindications	Few (e.g., iodinated contrast allergy)	Multiple (metallic implants, certain pacemakers, severe claustrophobia)
Suitability for emergency assessment	High	Low
Suitability for repeated/longitudinal imaging	Limited (radiation)	High

**Table 4:** Conceptual summary of the principal dimensions of sphenoid sinus morphology and their clinical and surgical implications.

Dimension	Key Findings	Clinical/Surgical Implication
Embryological development	Two-phase pneumatization (primary: birth–4 yrs; secondary: 4–16 yrs); stepwise bone marrow involution precedes air-cell expansion	Incomplete pneumatization in children limits transsphenoidal access; patient age is a critical planning variable
Pneumatization type	Five principal types (conchal to postsellar/clival); sellar type ~75%; population-level variation documented across multiple cohorts	Type determines working space, landmark availability, and neurovascular risk; individualised CT assessment mandatory
Intrasinus septation	Single midline septum in <40% of cases; deviated or multiple septa in majority; frequent termination on carotid sulcus or optic canal	Septal manipulation is a high-risk manoeuvre; preoperative mapping essential to avoid ICA or optic nerve injury
Neurovascular relationships	ICA dehiscence, optic nerve contact, reduced intercarotid distance; variable bony coverage of cranial nerves III–VI	Defines intraoperative risk; requires individualised MRI/CT/angiographic assessment prior to any transsphenoidal procedure
Radiological assessment	CT: gold standard for bony anatomy; MRI: gold standard for soft tissue; complementary and ideally co-registered	Integrated CT/MRI dataset is contemporary standard of care; angiographic techniques added when vascular pathology suspected
Emerging technologies	VR: preoperative patient-specific rehearsal and surgical training; AR: real-time intraoperative overlay of anatomical structures	Extend rather than replace conventional imaging; evidence base expanding but prospective outcome data still limited

## Data Availability

The data from which repositories the articles were found in has been published and is cited in the article.

## Abbreviations

The following abbreviations are used in this manuscript:

SS	sphenoid sinus (sinus sphenoidalis)
AI	artificial intelligence
VR	virtual reality
AR	augmented reality
CT	computed tomography
MRI	magnetic resonance imaging
CBCT	Cone Beam Computed Tomography
CTA	Computed Tomography Angiography
FESS	Functional Endoscopic Sinus Surgery
ICA	Internal carotid artery
HRCT	high-resolution computed tomography
CSF	cerebrospinal fluid
CN	cranial nerve(s)
PE	pterygoid recess
LE	lateral recess
